# Dropwise Condensation in Ambient on a Depleted Lubricant-Infused Surface

**DOI:** 10.1021/acsami.3c02450

**Published:** 2023-04-20

**Authors:** Durgesh Ranjan, Maheswar Chaudhary, An Zou, Shalabh C. Maroo

**Affiliations:** Department of Mechanical and Aerospace Engineering, Syracuse University, Syracuse, New York 13244, United States

**Keywords:** LIS, porous-nanochannels, oil-depletion, condensation, drop-distribution

## Abstract

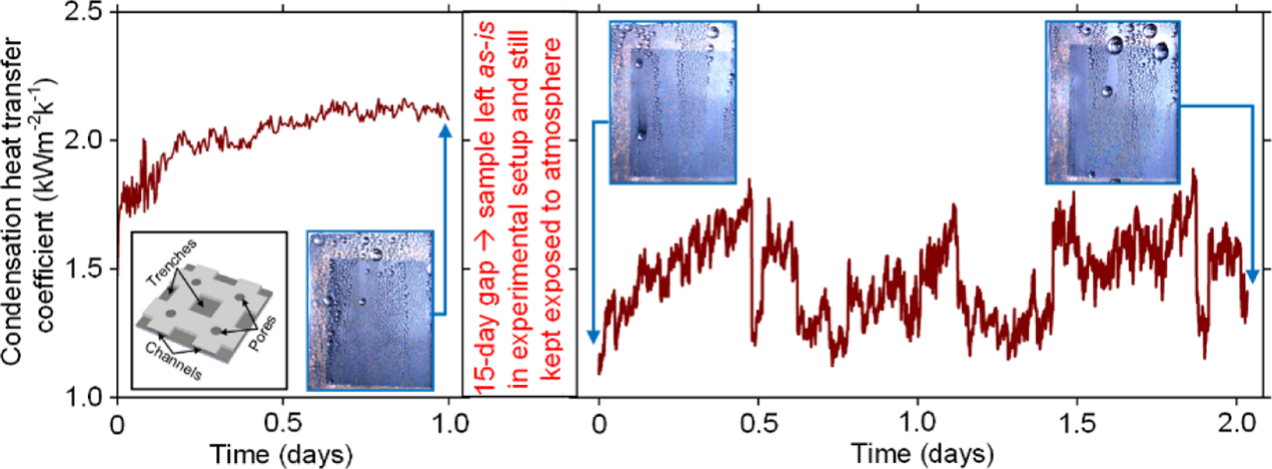

Durability of a lubricant-infused surface (LIS) is critical for heat transfer, especially in condensation-based applications. Although LIS promotes dropwise condensation, each departing droplet condensate acts as a lubricant-depleting agent due to the formation of wetting ridge and cloaking layer around the condensate, thus gradually leading to drop pinning on the underlying rough topography. Condensation heat transfer further deteriorates in the presence of non-condensable gases (NCGs) requiring special experimental arrangements to eliminate NCGs due to a decrease in the availability of nucleation sites. To address these issues while simultaneously improving heat-transfer performance of LIS in condensation-based systems, we report fabrication of both fresh LIS and a lubricant-depleted LIS using silicon porous nanochannel wicks as an underlying substrate. Strong capillarity in the nanochannels helps retain silicone oil (polydimethylsiloxane) on the surface even after it is severely depleted under tap water. The effect of oil viscosity was investigated for drop mobility and condensation heat transfer under ambient conditions, i.e., in the presence of NCGs. While fresh LIS prepared using 5 cSt silicone oil exhibited a low roll-off angle (∼1°) and excellent water drop (5 μL) sliding velocity ∼66 mm s^–1^, it underwent rapid depletion as compared to higher viscosity oils. Condensation performed on depleted nanochannel LIS with higher viscosity oil (50 cSt) resulted in a heat-transfer coefficient (HTC) of ∼2.33 kW m^–2^ K^–1^, which is a ∼162% improvement over flat Si-LIS (50 cSt). Such LIS promote fast drop shedding as is evident from the little change in the fraction of drops with diameter <500 μm from ∼98% to only ∼93% after 4 h of condensation. Improvement in HTC was also seen in condensation experiments conducted for 3 days where a steady HTC of ∼1.46 kW m^–2^ K^–1^ was achieved over the last 2 days. The ability of reported LIS to maintain long-term hydrophobicity and dropwise condensation will aid in designing condensation-based systems with improved heat-transfer performance.

## 1 Introduction

Surface modifications by fabrication of micro/nanotextured structures, followed by coating of low surface energy polymers (such as lubricants), can tailor the wetting or dewetting characteristics^1,2^ of surfaces. Taking inspiration from nature,^3,4^ numerous studies^5−8^ have combined engineered surfaces with lubricants to achieve a desired wettability. Such surfaces are often referred to as liquid-infused surfaces (LIS) and have been explored extensively owing to their excellent liquid repellency desired in numerous practical applications such as water harvesting,^9^ drag reduction,^10,11^ medical applications,^12^ corrosion resistance,^13,14^ anti-icing wind turbines,^15,16^ and condensation-based heat-transfer applications,^17^ among others. Excellent water repellency/hydrophobicity, low drop roll-off angle (ROA < 5°), and lower energy barrier for condensate nucleation^18^ make LIS suitable for condensation heat transfer. The superhydrophobic micro/nanotextured surface obtained from coating or depositing low surface energy polymers can exhibit a water contact angle (WCA) greater than 160° and roll-off angle (ROA) close to 1° accompanied by the Cassie–Baxter state in which the liquid drop rests on top of the rough surface without being wicked into the roughness.^19^ Condensing vapor on a pristine LIS exhibits extremely mobile and distinct liquid droplets (i.e., dropwise condensation or DWC), which get shed continuously resulting in significantly improved heat-transfer performance^20^ as compared to condensation on the plain surface over which a blanketing liquid film develops (i.e., filmwise condensation or FWC).

However, maintaining lasting DWC even on LIS is challenging without any external aid, as the droplet resting on the lubricant film gets cloaked and surrounded by lubricant wetting ridges,^21^ wherein oil rises up along the droplet’s outer contour causing oil depletion with each shedding condensate droplet.^22^ This is detrimental to the condensation performance of such surfaces which degrade rapidly in a highly humid environment due to rapid depletion of lubricant, causing delayed departure of multiple coalesced droplets. Eventually, drop pinning on the exposed rough surface is observed, and gradual flooding of the surface texture triggers undesired transition from the Cassie–Baxter state to Wenzel state, where a condensing drop permeates into the roughness underneath.^19^ Studies on LIS demonstrating long-term hydrophobicity and steady heat-transfer performance are lacking. LIS can be designed such that during condensation, small condensate droplets depart before undergoing multiple coalescence thus achieving high heat-transfer coefficients,^23^ as the span of nucleation-to-departure is shorter resulting in a higher fraction of fresh sites available for continuous nucleation. This feature of the faster condensate drop departure has been found to be unsustainable for longer periods of time in reported studies. Additionally, heat-transfer performance deteriorates^24,25^ significantly when condensation is performed in the presence of non-condensable gases (NCGs) or in an open environment, as gases act as a thermal barrier and occupy potential nucleation sites on the lubricant–vapor interface, which explains the relatively few studies^25−27^ reporting condensation in the presence of NCGs. Performing condensation without NCGs also requires additional equipment and design considerations^17,18,22,27−29^ such as specialized chambers and vacuuming systems to ensure the absence of NCGs at the lubricant–vapor interface. Thus, condensation on LIS without the need of active lubricant feeding mechanisms and having adequate heat-transfer performance is desired even in the presence of NCGs.

A typical LIS has an underlying micro/nanotextured surface coated with low surface energy polymer/chemical, which then gets infused with non-toxic, low surface tension, and low vapor pressure lubricants.^26−32^ Several techniques, such as gravimetric draining,^34^ spin-coating,^35^ or dip coating,^36^ are used to obtain a desired thickness of lubricant film on the surface. However, functionality of all these surfaces relies on the retention of oil in the surface topography either through active lubricant supply^37^ or by a special design arrangement which can continuously feed the textured surface.^38^ Apart from wetting ridges and condensate cloaking-induced depletion,^39^ LIS undergo external shear^40^-driven drainage during relative motion between the LIS and any fluid. Consequently, numerous fabrication techniques have been reported to mitigate lubricant depletion and to extend the operational lifespan of LIS, such as, utilizing metal oxide nanowires and pores,^9,13^ trapped air,^11^ and self-functionalizing lubricants,^41^ among others. However, after prolonged use of LIS, the lubricant layer on such surfaces depletes, causing drop pinning and decline in wetting behavior. Depletion is even more pronounced in condensation-based applications as lubricant layer thickness is small and usually dictated by an acceptable limit of oil contaminants in condensates. Thus, efforts are required to sustain the condensation heat-transfer performance on LIS for a prolonged duration, i.e., have a surface that would perform adequately even in depleted conditions as well as in an open environment (i.e., presence of NCGs).

Herein, we present a systematic approach to develop LIS using the following: (1) a hydrophilic porous nanochannel wick fabricated in a 500 μm thick silicon wafer and (2) commonly used non-toxic silicone oil (polydimethylsiloxane; a liquid siloxane) (viscosity η_o_ = 5, 50, 500, 1000 cSt) as a lubricating medium. There is neither any additional functionalization apart from the oil infusion nor any active oil feed arrangement. The infusion of silicone oil in a plasma-treated porous nanochannel wick substrate generates hydrophobic LIS with an apparent water contact angle of ∼102^°^. Furthermore, freshly prepared nc-LIS (nanochannel-LIS) shows excellent drop mobility, as is evident from small ROA∼ 1^°^ and a high-water droplet sliding velocity (*V*_S_) of ∼66 mm s^–1^ (drop size ∼5 μL, η_o_ ∼ 5 cSt, and inclination ∼25°). Depleted nc-LIS (dep-nc-LIS) was obtained by subjecting the surface to 4 h of gravimetric depletion followed by tap water jet (velocity ∼0.4 ms^–1^) shear depletion^40,42^ for 20 min. Significant enhancement in the condensation heat-transfer coefficient (HTC) (average HTC ∼2.33 kW m^–2^ K^–1^ with 50 cSt viscosity oil) was obtained on dep-nc-LIS as compared to freshly prepared LIS reported in the literature^27,43−47^ (HTC ∼0.40–2 kW m^–2^ K^–1^) in the presence of NCGs for condensation performed at atmospheric pressure (average relative humidity ∼87%, i.e., vapor mass fraction ∼78 gm of water per kg of dry air). Furthermore, HTC obtained from dep-nc-LIS shows ∼162% improvement compared to fresh LIS prepared on flat silicon wafer using 50 cSt silicone oil. The image analysis of condensate droplets during condensation reveals lower water coverage on the surface for dep-nc-LIS with 50 cSt oil at any moment, which explains the faster departure of the condensate drops. In addition to the reaction of silicone oil polymer chain (polydimethylsiloxane) with an inorganic silicon substrate,^48^ improvement in the heat-transfer coefficient can be attributed to strong capillarity inside nanochannels to retain oil even after undergoing severe gravity and shear depletion, thus maintaining its hydrophobic property (WCA >100° for all four viscous oils on depleted nc-LIS). To observe the long-term performance, condensation experiments were also performed for 3 days on fresh nc-LIS to observe the HTC variation with oil depletion. HTC showed an improvement of 16% due to initial oil depletion over the first 24 h (2.12 ± 0.02 Wm^–2^ K^–1^). The nc-LIS achieved steady HTC ∼1.46 kW m^–2^ K^–1^ in the last 2 days of condensation while maintaining dropwise condensation and hydrophobicity (WCA∼104°), which is similar to HTC values in the presence of NCGs reported with fresh LIS. Because the LIS fabrication in the current study does not include any additional functionalization before lubricant infusion, the prepared surface has shown the ability to regain its original hydrophilicity of dry sample (before oil infusion) by undergoing a standard cleaning procedure using petroleum ether, IPA, acetone, and plasma cleaning. Additionally, as nc-LIS presented here does not show degradation of underlying porous nanochannel geometry due to jet impact/shear-induced depletion and cleaning procedure, such unique regenerative capability could be convenient in applications involving multiple usage of the same sample.

## 2 Experimental Section

### 2.1 Nanochannel Wick Fabrication Process

Fabrication of porous nanochannel sample^49,50^ on a 500 μm thick silicon wafer involves dedicated photolithography procedures using a sacrificial chromium and copper layer. The detailed procedure and methodology are provided in the Supporting Information (Figure S1 and Supporting Note 1). Nanochannels fabricated orthogonally on silicon wafer have both width and pitch of ∼5.68 μm and a height of ∼729 nm [Figure S2(a–d)] creating numerous intersections and trenches. At each intersection, a pore of diameter ∼2 μm was fabricated, which allows oil to wick into the nanochannels. The porosity of nanochannel sample used in the current study is ∼0.75.

### 2.2 Materials and LIS Preparation Procedure

Acetone (Sigma-Aldrich, USA, CAS: 179124), ethanol (Sigma-Aldrich, USA, CAS: 362808), and petroleum ether (Sigma-Aldrich, USA, CAS: 184519) were used as chemical cleaning agents. Silicone oil of four different viscosity: 5 cSt (Sigma-Aldrich, USA, CAS: 317667), 50 cSt (Sigma-Aldrich, USA, CAS: 378356), 500 cSt (Sigma-Aldrich, USA, CAS: 378380), and 1000 cSt (Sigma-Aldrich, USA, CAS: 378399) were used in preparation of LIS. Physical properties of lubricants^33,51^ are presented in Supporting Information, Table S1. Fluorescein sodium salt (Sigma-Aldrich, USA, CAS: 46960) was used to trace the droplet path to obtain sliding velocity. Ethylene glycol-deionized water 50/50 blend (Cole Parmer, USA, Item: EW-78930-17) was used as cooling fluid in a recirculating chiller during condensation experiments. LIS preparation starts with cleaning the nanochannel sample chemically followed by 10 min of high-power plasma cleaning (Harrick Plasma, USA, Model: PDC-001-HP). The sample was subsequently flooded with silicone oil. The sample was then placed in a convective oven (MTI corporation, USA, model: DHG-9070AS) overnight at 150 °C for baking. This process of baking was found to be beneficial in keeping the surface hydrophobic and ensuring proper infusion of oil in the channels even for high viscosity oil. The flooded sample undergoes gravimetric depletion for 4 h resulting in the fresh nanochannel LIS (nc-LIS) sample, which was also used in measuring water drop sliding velocity. To attain a depleted nanochannel LIS sample (dep-nc-LIS), nc-LIS was kept horizontally under a tap water jet to further deplete oil under shear imparted by running tap water (jet velocity ∼0.4 ms^–1^) for 20 min from a nozzle of diameter 4.6 mm (Supporting Movie S1), followed by 30 min in a convection oven at 150 °C. Keeping the sample in the oven is important as the presence of water would hamper the weight measurement of depleted LIS samples to obtain the amount of oil present in the sample. Another reason to keep the sample in an oven was to have uniform contact angles at various locations on the surface by inducing uniform oil infusion and baking.

### 2.3 Characterization

Micrographs of nanochannel sample were captured using an upright microscope (Nikon, Model: Eclipse-LV150NL). Surface topography of sample was analyzed via atomic force microscopy (AFM) using Vecco Icon AFM tool. A Bruker hyperion FT-IR spectrometer was used to obtain a FT-IR spectrum of dep-nc-LIS samples. Weight measurements were taken by using a Pioneer analytical (Ohaus, USA, model: PX224/E, least count: 0.1 mg) weighing scale. All contact angle measurements were repeated six times in ambient conditions (23 °C, 40% relative humidity) with a water drop volume of 3 ± 1 μL on a VCA optima goniometer. Accuracy of goniometer tool is 1°. A high-speed camera (Phantom, USA, model: V611) captured water droplet motion, which was later used to obtain sliding velocity by performing image analysis through a custom MATLAB script. The temperature was recorded using combination of K-type thermocouples (Omega, USA, model: SCAXL-020-6) and T-type thermocouples (Omega, USA, model: SCPSS-020-6) connected to a data acquisition system (National instruments, USA, NI 9211).

### 2.4 Condensation and Drop Size Analysis

Condensation was performed on LIS in the presence of non-condensable gases. The condensation chamber was open to the atmosphere with average relative humidity inside the chamber being ∼87%. The backside of LIS was attached to an aluminum cold plate through which thermal fluid (ethylene glycol-deionized water mixture) was allowed to flow. The temperature of thermal fluid was controlled by a recirculating chiller (Cole-Parmer, USA, Model: Polystat CR250WU) and flow rate was maintained by a custom-built flow rate control circuit. Condensation chamber humidity was monitored by using 2-Channel compact USB temperature and humidity logger (ThorLabs, USA, Model: TSP01). A video camera was used to record the condensate drop size distribution at different time intervals. These recorded frames were then analyzed for the droplet size distribution through a custom written MATLAB script. Portions of original recorded images are selected based on the clarity and brightness to minimize error during analysis. Drops are converted into the binary form by choosing a threshold value of inbuilt MATLAB function parameters followed by creating circles encapsulating individual droplets. Pixels corresponding to each drop in the images give the respective diameter.

## 3 Results and Discussion

### 3.1 Surface Morphology and LIS preparation

**Figure 1:**
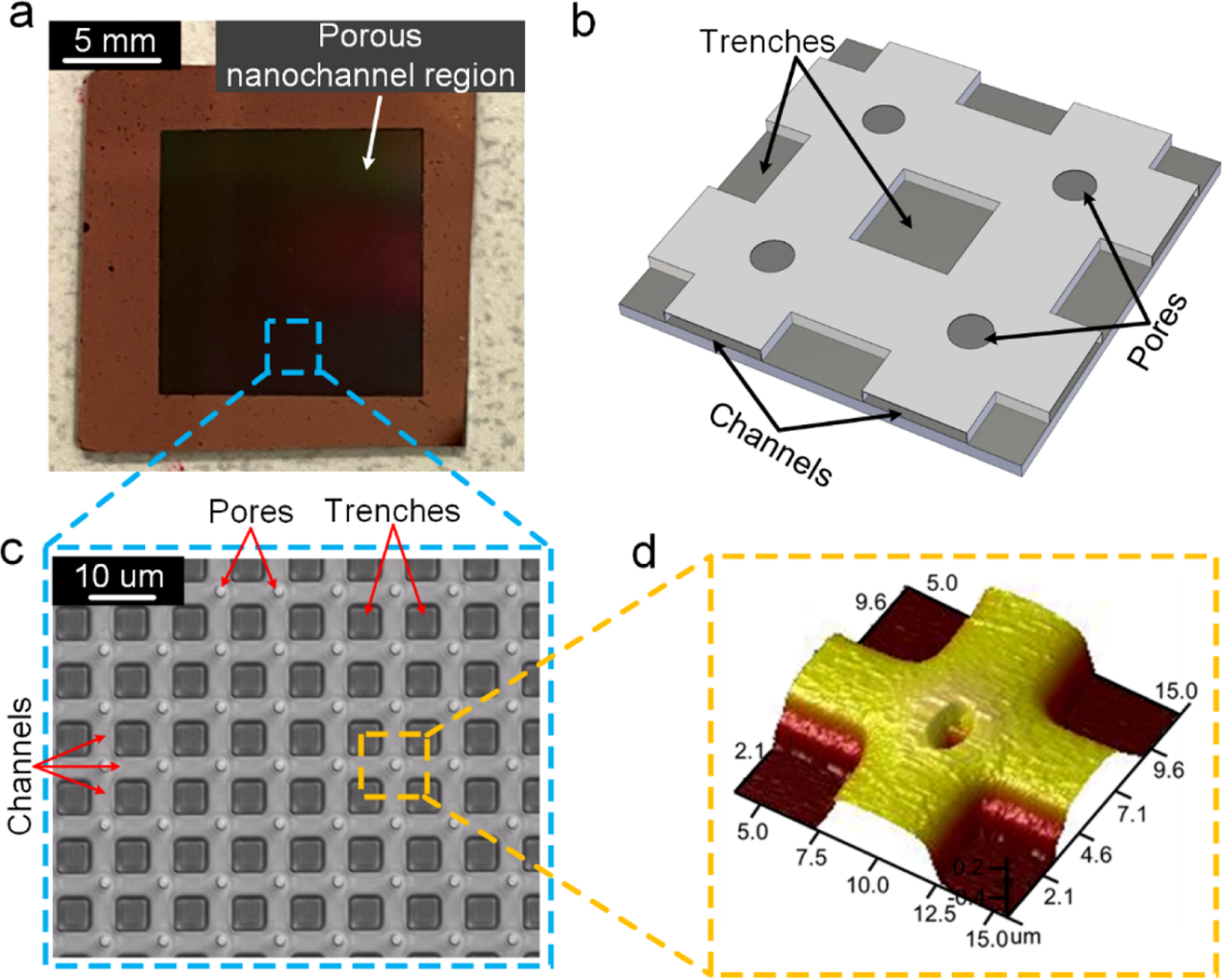
Sample details, morphology characterization, and LIS preparation: (a) silicon base showing a nanochannel porous region, (b) three-dimensional schematic of a sample unit cell in the porous region comprising intersecting channels (height ∼729 nm, width ∼5.68 μm), four pores (diameter ∼2 μm) at each channel intersection, and trenches, (c) micrograph of porous nanochannel sample, and (d) AFM image of a pore fabricated at the channel intersection.

Figure 1a shows the nanochannel sample used in the current study with a hydrophilic (WCA ∼10 °C) porous nanochannel region. The porous region is 14 mm by 14 mm and the surrounding surface on the outside is flat silicon wafer. The three-dimensional schematic (Figure 1b) shows interconnected nanochannels where a pore of ∼2 μm diameter has been fabricated at each intersection, allowing the lubricant to wick into the nanochannels (height ∼729 nm, width ∼5.68 μm). Details on surface morphology can be found in micrographs and AFM of the sample, as presented in Figures 1c and 1d, respectively.

**Figure 2:**
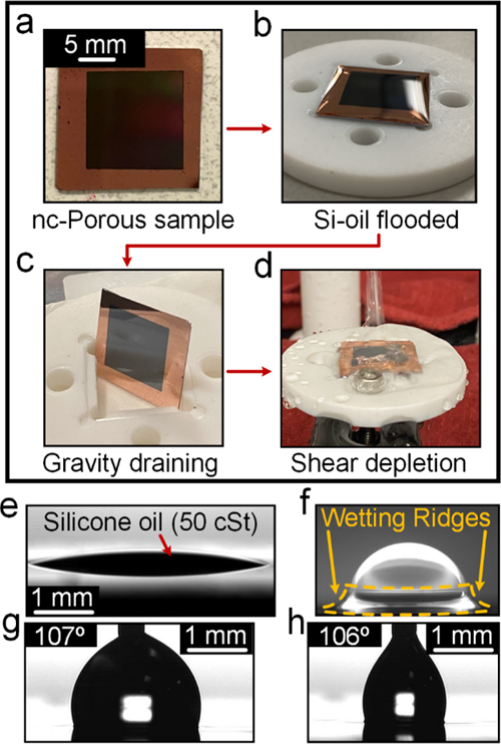
Preparation of liquid-infused surface: (a–d) nanochannel-depleted lubricant-infused surface preparation stages: a pristine sample followed by flooding with silicone oil, gravimetric depletion and shear depletion under tap water at 0.4 ms^–1^, (e) snapshot of silicone oil (50 cSt) spreading on the porous nanochannel wicks sample, (f) wetting ridges around fluorescence dissolved-water drop on (nanochannel) nc-LIS 50 cSt, and (g–h) advancing and receding contact angles on nc-LIS 5 cSt, respectively.

The variation in the nanochannel height across the nanochannel width obtained from AFM at multiple locations on the sample to confirm the dimensions is shown in Figure S2a–d. Figure 2a through 2d depicts the preparation of nc-LIS, starting from flooding the plasma-treated nanochannel sample with adequate silicone oil (Figure 2b), baking in a convective oven overnight at 150 °C, followed by 4 h of oil draining under gravity (Figure 2c), resulting in the fresh nc-LIS surface. Further depletion of oil under tap water jet (Figure 2d) results in depleted nc-LIS. An image captured during spreading of silicone oil drop (η_o_ = 50 cSt) on the plasma-treated nanochannel sample is shown in Figure 2e. Such four different nc-LIS samples were obtained using different silicone oils (viscosity η_o_ = 5, 50, 500, and 1000 cSt; for reference DI water has viscosity 1 cSt). Roll-of-angle (ROA) measured with 5 μL water drop using a tilting table (least count ∼ 1°) for nc-LIS surfaces prepared from 5, 50, 500, and 1000 cSt were ∼1, ∼1, ∼4, and ∼6° respectively. High viscosity accounts for increased friction at the drop–oil interface during sliding, resulting in the observed increment in ROA with oil viscosity.^52^ Oil thickness (*t*_o_) on the surface of fresh nc-LIS was obtained using a mass change technique^9,53^ using the following equation
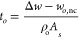
1where Δ*w* is the difference between weights of dry sample (*w*_dry_) measured before oil infusion and weight after gravimetric draining (*w*_g_) and (*w*_o,nc_) is the theoretical weight of oil in present within the channels and pores (∼0.1 mg), ρ_o_ is the oil density, and *A*_s_ is the projected area of the porous region of the sample. *w*_o,nc_ is obtained from the volume of the nanochannels and the density of silicone oil. Oil thickness considering the least count of the weighing scale (0.1 mg) was obtained to be 1.29 ± 0.56 μm, 4.01 ± 0.54 μm, 7.47 ± 0.53 μm, and 10.04 ± 0.53 μm, respectively, with increasing viscosity of oils.

Further, a freshly prepared nc-LIS surface was found to exhibit wetting ridges as seen around a fluorescence water drop (Figure 2f) similar to numerous reported studies.^6,21,22,29,54^ Despite being not clearly visible from Figure 2f, theoretical prediction criteria given in eq 2 suggests the presence of cloaking around the drop, as shown in Figure 2f.
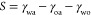
2where *S* is the spreading coefficient (*S* > 0 signifies cloaking), γ_wa_ = 72.7 m Nm^–1^ is the interfacial tension between water and air, γ_ow_ = 6 m Nm^–1^ is the interfacial tension between oil and air,^21^ and γ_oa_ = 21 m Nm^–1^ is the interfacial tension between water and oil. A positive value for the spreading coefficient is obtained for all four-silicone oil used in the present study. Water contact angle hysteresis (CAH) measured on freshly prepared nc-LIS with 5 cSt oil shows very low difference (∼0.6°) between advancing and receding contact angles (Figure 2g,h), which is a desired characteristic of any LIS. Tap water jet (Figure 2d) on the nc-LIS sample was used to obtain the dep-nc-LIS sample, as explained in section 2.2. Although shear imparted by flowing water did result in severe depletion of oil, all the surfaces remained hydrophobic with WCA >100°. In addition to the reported studies^55−57^ on the plasma-treated surface, activating enhanced hydrophilization and improving adhesive strength of siloxane, strong capillarity within the nanochannels and micropores appears to be responsible for retaining oil and thus sustaining surface hydrophobicity even after severe water shear depletion.

### 3.2 Effect of Viscosity on Drop Mobility

**Figure 3:**
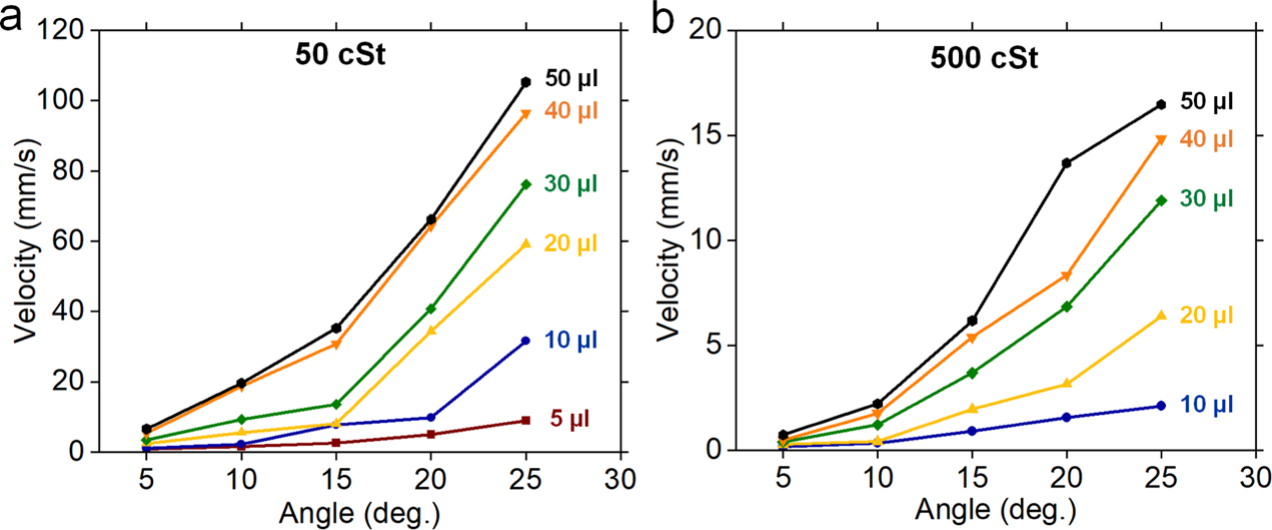
Sliding velocity (*V*_s_) for water drop of various volumes on the freshly prepared nanochannel lubricant-infused surface (nc-LIS): (a–b) variation of water (volume: 5–50 μL) droplet velocity for different angles of inclination and prepared nc-LIS with different silicone oil viscosity (50, 500 cSt).

Freshly prepared nc-LIS for all four-silicone oil viscosities were subjected to the water drop mobility test by measuring the velocity of various drop sizes at multiple angles of inclination. Water drops (with dissolved fluorescence salt) sliding down the nc-LIS prepared with 5 cSt silicone oil are kept at 5° inclination is shown in Figure S3. An image processing algorithm, which tracks the drop contour during motion is implemented to determine the average drop velocity with very high accuracy (Supporting Note S3, Figure S3). Variation of water drop sliding velocity (V_s_) on nc-LIS with a surface angle of inclination for different drop volumes (5–50 μL) for oil viscosity: 50 and 50 cSt is shown in Figure 3a,b; while for oil viscosity: 5 and 1000 cSt, it is shown in Figure S4a,b. Similar to previously reported studies,^27,52,58^*V*_s_ is observed to be inversely proportional to oil viscosity and increases with both angles of inclination and drop volume. Thus, as far as drop mobility is concerned in the freshly prepared state, nc-LIS behaves similar to the reported LIS surfaces. *V*_s_ obtained on nc-LIS with 5 cSt oil was an order of magnitude higher than those observed with 500 cSt or 1000 cSt oils. Although high *V*_s_ associated with less viscous lubricants is a desirable characteristic, it undergoes rapid oil depletion as reported in the next section. Therefore, choosing suitable viscosity lubricants becomes critical in maintaining durability of LIS properties.

### 3.3 Effect of Viscosity on Lubricant Depletion

**Figure 4:**
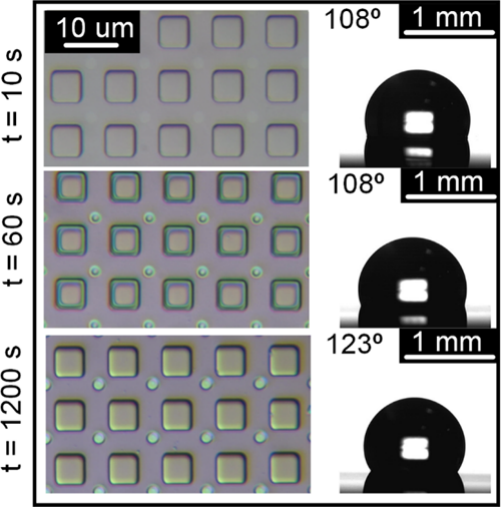
Effect of water shear depletion as noticeable from micrographs and water contact angle measurements at different time intervals.

**Figure 5:**
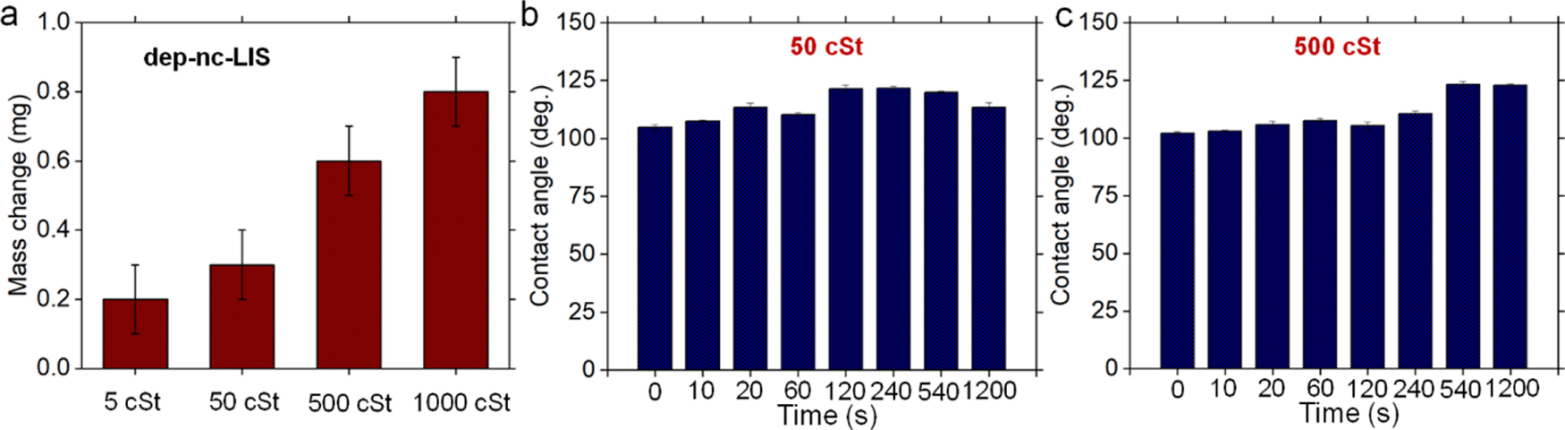
Effect of water shear depletion on the wettability of nanochannel depleted the lubricant-infused surface (dep-nc-LIS): (a) difference in the weight of dry sample (before oil infusion) and dep-nc-LIS surface for prepared nc-LIS with different silicone oil viscosities (5, 50, 500, and 1000 cSt) and (b,c) variation in water contact angles observed during tap water shear depletion of fresh nc-LIS for prepared samples having different silicone oil viscosity (50, 500 cSt).

Figure 4 shows changes in the surface appearance under a microscope with time of shear depletion under tap water jet and their respective WCA. The presence of the silicone oil in dep-nc-LIS, following water shear depletion (Figure 2d), was confirmed by quantifying the weight change (Δ*w*_d_, Figure 5a) given by the following equation
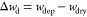
3where *w*_dep_ is the weight of dep-nc-LIS sample and *w*_dry_ is the weight of dry sample measured before oil infusion. The silicone oil retention in nanochannels can be attributed to the following: (1) capillarity due to open pores and (2) combined effect of improved adhesion^48^ (of silicone oil due to oxygen plasma cleaning), or any other intermolecular interaction^59^ between the surface and the oil. The effect of individual factors mentioned have been found to enhance the oil retention in the nc-LIS. FT-IR (Figure S2e, Note S2) spectrum of dep-nc-LIS surfaces confirms the presence of C–H, Si–O–Si present in silicone oil even after 20 min of shear depletion under tap water. Oil thickness in dep-nc-LIS, following the water jet shear-induced depletion was found to be ∼245, ∼601 nm, ∼1.36, and ∼2.11 μm for 5, 50, 500, and 1000 cSt, respectively, as evaluated using eq 1.

Detailed temporal variation of apparent WCA due to shear depletion for 50 and 500 cSt oil samples is presented in Figure 5b,c while for 5 and 1000 cSt, it is shown in Figure S5. While apparent WCA increased at first, depletion of oil is found to be relatively higher^40,60^ in low viscous lubricants based on the observed decline in apparent WCA after ∼240 s for 5 cSt (Figure S5) due to droplet pinning, and ∼540 s for 50 cSt due to a higher contact angle hysteresis (Figure 5b). With gradual depletion of oil, contact line concealed within the lubricating meniscus progressively becomes observable and, LIS moves from excess-oil to oil-starved state, thus shifting the observable water drop contact line close to the true contact line on the surface (unaffected by oil ridges) and hence, the measured WCA is observed to increase^61^ for all four cases up to a certain duration depending on oil viscosity. Conversely, 500 cSt (Figure 5c) and 1000 cSt (Figure S5) oil samples show an increment in the WCA till 1200 s as the depletion process is ongoing, as evident from the more oil retention on such surfaces (Figure 5a). While both surfaces with 500 and 1000 cSt oil possess the desired wettability, drop mobility on these surfaces is found to be unfavorable (Figures 3b, S4b). Thus, dep-nc-LIS with 50 cSt oil presents a good trade-off between both features, i.e., high drop mobility and persistent hydrophobicity during depletion, making it a suitable choice for evaluating its condensation heat-transfer performance. In the present study, based on *V*_s_ and oil depletion (inferred from oil mass change, Figure 5a), we found nc-LIS prepared with 50 cSt oil performed superior to nc-LIS prepared with other viscosity oils.

### 3.4 Condensation on the Porous Nanochannel Lubricant-Infused Surface

**Figure 6:**
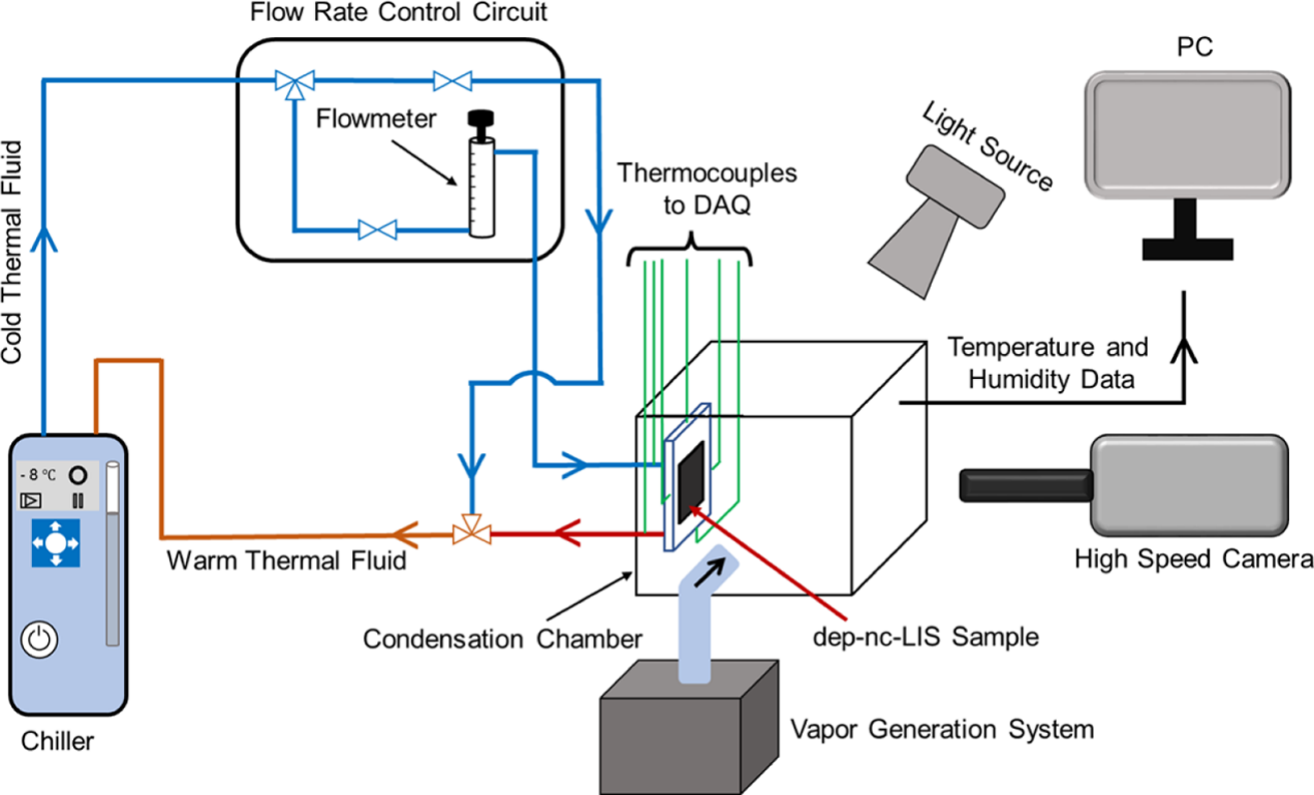
Schematic of custom-built condensation experiment setup.

In order to test the heat-transfer performance of our LIS, all condensation experiments were carried out in ambient air, i.e., in the presence of non-condensable gases (NCGs). A schematic of the experimental setup is shown in Figure 6. To conduct the condensation experiments, we chose a nanochannel sample with a larger sample size (28.1 mm by 35.8 mm) but with the same underlying geometry dimension of nanochannels and micropores. After sample preparation and oil depletion, LIS was attached to a custom-made aluminum plate through which cold thermal fluid (ethylene glycol-water) was supplied from a recirculating chiller. A constant flow rate of cold thermal fluid was maintained using a flow meter valve in a custom-designed flow rate control circuit. The aluminum cold plate was sealed on all exposed areas using the RTV silicon sealant except for the LIS sample. The cold plate along with the sample was subsequently positioned on one of the faces of an open condensation chamber facing a high-speed camera (Figures 6, S6). Four thermocouples were inserted (3 mm under the condensing LIS surface from all four sides) in the cold plate to record the surface temperature. Moreover, two thermocouples were inserted in the inlet and outlet tubes of cold plates (Figure S7) to monitor the temperature change (Δ*T*_w_) of water-glycol mixture during condensation that was further used in condensation heat-transfer (*Q*_w_) calculations. Effect of the thermal boundary layer on the temperature of the thermal fluid at the outlet was investigated and found to be insignificant (Figure S8, Note S4). Humidity and temperature in the condensation chamber was maintained by a boiling-based external vapor generation system.

Heat-transfer coefficient (HTC) calculation involves the following two equations
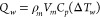
4
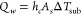
5where *Q*_w_ is the condensation heat transfer (*W*) and is assumed to be the same as heat taken away by cold thermal fluid, ρ_m_ = density of water-glycol mixture (50:50), 1085 kgm^–3^ at 10 °C, *V*_m_ is the volume flow rate of water-glycol (50:50) during the experiment (47.7 ccm), *C*_*p*_ is the specific heat of water-glycol (50%), 3.44 kJ kg^–1^ K^–1^at 10 °C, h_c_ is the condensation heat-transfer coefficient (Wm^–2^ K^–1^), *A*_*s*_ is the LIS projected surface area, 1005.98 × 10^–6^ m^2^, and Δ*T*_sub_ is the temperature difference between dep-nc-LIS average surface temperature and ambient condensation chamber temperature (*T*_amb_). Calculations for *h*_*c*_, *Q*_*w*_, and related error and uncertainty analysis are presented in Supporting Information (Supporting Note S5, Table S2). Two different sets of condensation experiments were performed. In the first set of experiments that lasted for 4 h and 15 min each, flat Si-LIS and depleted nc-LIS (50, 500 cSt) samples were used. Results obtained from above experiments were evaluated against the second type of experiment, which involved 3 days of condensation performed over an 18 day period on fresh LIS on porous nanochannels with 50 cSt silicon oil, referred to as nc-LIS-50. Similarly, samples of dep-nc-LIS with 50 cSt oil and 500 cSt oil will be referred to dep-nc-LIS-50 and dep-nc-LIS-500, respectively, in the remainder of this work.

**Figure 7:**
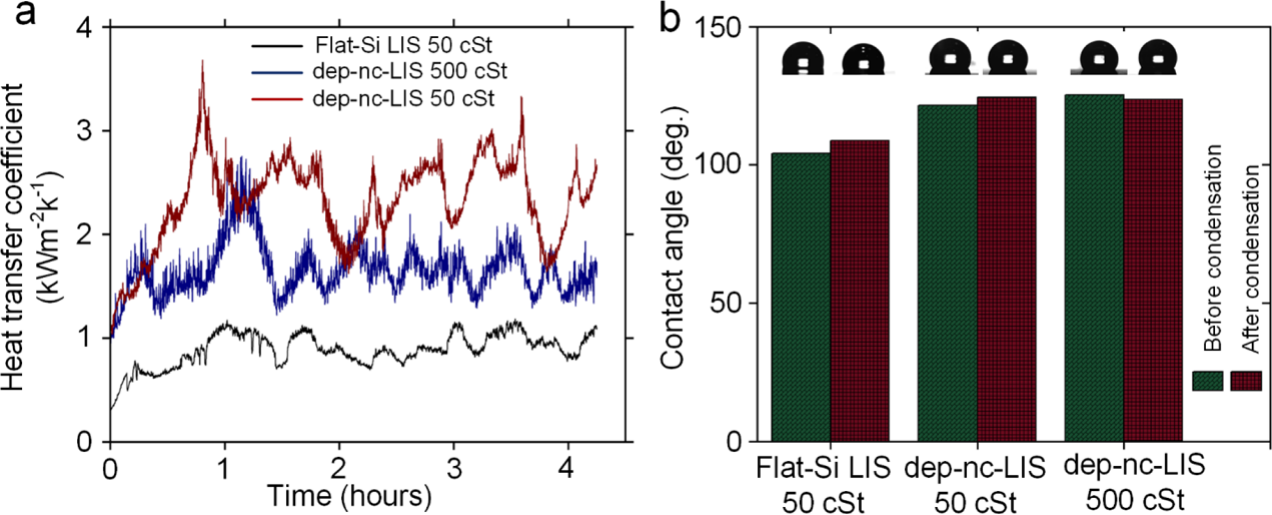
Condensation of water vapor on three different depleted liquid-infused surfaces, namely, flat Si-LIS with 50 cSt oil (LIS on the flat silicon surface), and two nanochannel depleted lubricant-infused surfaces (dep-nc-LIS with 500 cSt oil, and dep-nc-LIS with 50 cSt oil), showing: (a) variation of heat-transfer coefficient (HTC) for depleted samples and (b) water contact angle before condensation and after 4 h 15 min of condensation on the depleted sample.

For the first set of experiments conducted for 4 h and 15 min each, variation of HTC for flat Si-LIS with 50 cSt oil, dep-nc-LIS-50, and dep-nc-LIS-500 is shown in Figure 7a. Variation of ambient chamber temperature (*T*_amb_), average sample temperature (*T*_sample,avg_), thermal fluid temperature at the cold plate outlet (*T*_fluid,out_), and thermal fluid temperature at the cold plate inlet (*T*_fluid,in_) during condensation is shown in Supporting Information, Figure S9. Although there are localized variation in HTC throughout the experiment (due to varying degrees of vapor content coming into the condensing chamber, which dictates the Δ*T*_sub_, overall HTC obtained for dep-nc-LIS-50 was as follows: 2.33 ± 0.42 Wm^–2^ K^–1^, which is ∼162 and ∼40% improvement over flat Si-LIS with 50 cSt oil (*h*_c_ = 0.89 ± 0.16 Wm^–2^ K^–1^) and dep-nc-LIS-500 (*h*_c_ = 1.66 ± 0.25 Wm^–2^ K^–1^), respectively. In the case of flat Si-LIS with 50 cSt oil, the depletion process preceding condensation resulted in the removal of surface oil to the fullest extent as is apparent from the temporal drop size distribution during condensation [Figure S10(i–p)]; hence, flat Si-LIS with 50 cSt oil exhibits lower HTC over time. Moving average of HTC for dep-nc-LIS-50 and dep-nc-LIS-500 reveals modest depreciation as is evident from the small increment in the condensate drop size on the surface and delayed shedding. While condensing surface being in the depleted state does not hold sufficient oil to cloak droplets, there exists the possibility of oil depletion (during droplet shedding) from shear due to oil menisci formed at ridges. This shear depletion of oil is opposed by the existing capillary pressure inside the nanochannels and is inversely proportional^40^ to oil viscosity, thus causing retention of oil on the sample. Variation in surface wettability because of condensation is reported in the form of WCA measured before and after condensation experiments for all three cases (Figure 7b). Maximum variation was found to be ∼ 5° for the flat Si-LIS surface and was less than ∼3° for dep-nc-LIS surfaces.

**Figure 8:**
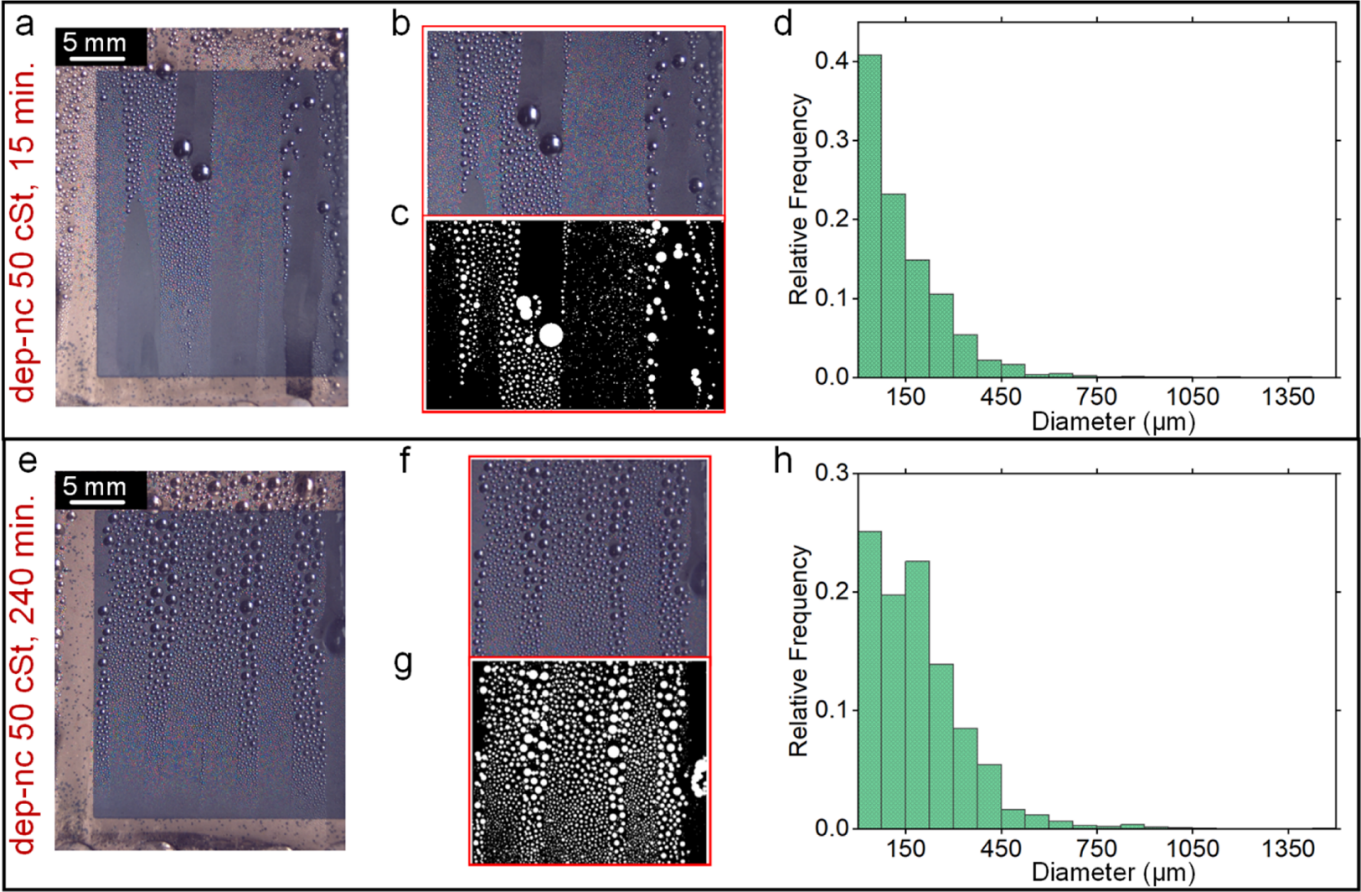
Drop size distribution for a selected region on the nanochannel depleted liquid-infused surface (dep-nc-LIS) samples during the experiment for dep-nc-LIS with 50 cSt oil after (a–d) 15 min and (e–h) 240 min of condensation.

A distinct change in the contact angle hysteresis (CAH) of all three samples used for condensation can be seen in Table 1. Considering severe depletion of oil before condensation, droplet pinning on flat Si-LIS with 50 cSt oil is obvious. Even though dep-nc-LIS-50 and dep-nc-LIS-500 did not manifest droplet pinning, a significant rise in CAH after condensation indicates surface degradation. The extent of surface deterioration during the condensation experiment for flat Si-LIS with 50 cSt oil (Note S6, Figure S10(i–p)), dep-nc-LIS-50 (Figure 8a–h), and dep-nc-LIS-500 (Figure S10(a–h)) has been investigated by analyzing the condensate drop size distribution on the condensing surfaces (Figure 8d,h). Histograms (bin size = 75 μm) show the relative frequency of occurrence of a particular size group of droplets. Drop growth on the upper portion of the sample is faster as cold fluid from a chiller enters at the top of cold plate, prompting coalescence and earlier shedding of drops there than those at the bottom of surface. The departing drop leaves behind a regenerated area for re-nucleation of new drops. Consequently, frequent drop departure results in a higher fraction of condensation surface observing nucleation and hence, an improvement in HTC. It was found that the percentage of drops having diameter <250 μm at the beginning of condensation was ∼41, ∼84, and ∼65% for flat Si-LIS, dep-nc-LIS-50, and dep-nc-LIS-500, respectively, which dropped toward the end of condensation to ∼28, ∼73, and ∼52%, respectively, for the same surfaces. Throughout the condensation, the fractional coverage of large drops is most pronounced in the flat Si-LIS surface and is undesired because water has high conduction resistance during high heat transfer between hot vapor and condensing surfaces. This is consistent with the obtained HTC for all three surfaces. A comparison of drop size <500 μm elucidates the contrasting difference in heat-transfer performance of dep-nc-LIS surfaces compared to the flat Si-LIS surface. For dep-nc-LIS-50, percentage of drops having diameter <500 μm decreased from ∼98% (at the beginning of condensation) to only ∼93% (toward the end of condensation) and for dep-nc-LIS-500 from ∼91 to ∼82%. As expected, the reduction was more prominent for flat Si-LIS (from ∼80 to ∼61%).

**Table 1:** Variation in the Contact Angle Hysteresis after Condensation

	before condensation			after condensation		
depleted LIS surface type	advancing 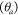	receding 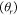	hysteresis 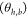	advancing 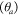	receding 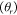	hysteresis 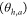
flat Si-LIS-50 cSt	pinned	pinned	N/A	pinned	pinned	N/A
dep-nc-LIS-50	117	88	29	126	72	54
dep-nc-LIS-500	124	93	31	127	71	56

**Figure 9:**
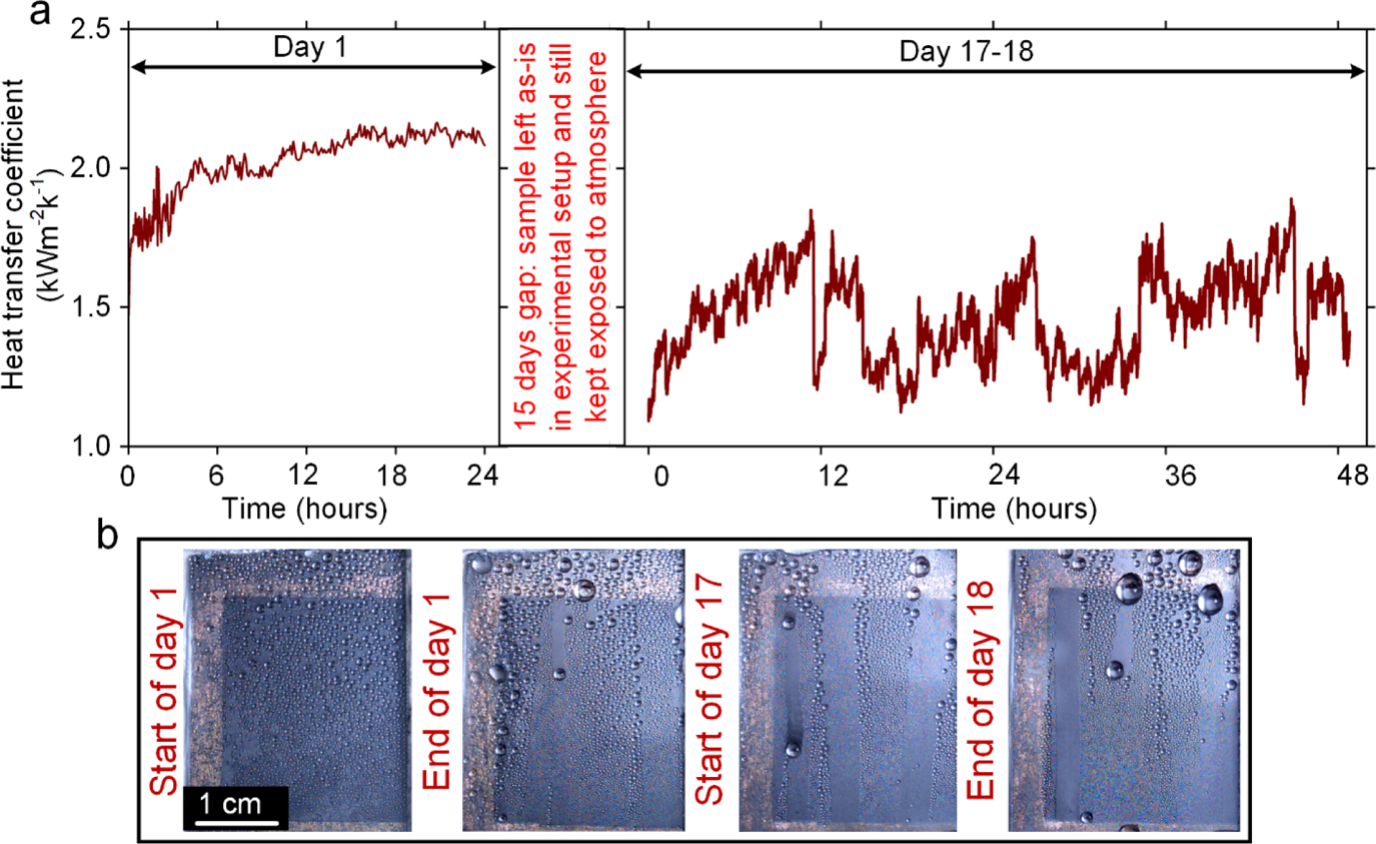
Condensation of water vapor on freshly prepared liquid-infused surface on the porous nanochannel with 50cSt silicone oil: (a) variation of heat-transfer coefficient (HTC) for a total of 3 days of condensation over an 18 day period and (b) drop visualization at different times during the condensation experiment.

For the second set of experiments, condensation on fresh LIS on the porous nanochannel sample with 50 cSt silicon oil (nc-LIS-50) was conducted for a total of 3 days in two phases over a period of 18 days. The first phase of the experiment included continuous condensation over the first day (24 h), followed by 15 days of keeping the sample as-is in the experimental setup and still under ambient conditions. The second phase involved 2 days (48 h) of continuous condensation on the same nc-LIS-50 sample. Heat-transfer coefficient variation for nc-LIS-50 is shown in Figure 9a and respective variation of ambient chamber temperature (*T*_amb_), average sample temperature (*T*_sample,avg_), thermal fluid temperature at the cold plate outlet (*T*_fluid,out_), and thermal fluid temperature at the cold plate inlet (*T*_fluid,in_) during condensation is shown in Supporting Information, Figure S11. The average heat-transfer coefficient in the first 4 h of experiments was: 1.83 ± 0.11 Wm^–2^ K^–1^, which is similar to the reported values^27,45,47^ for condensation in the presence of NCGs. However, variation in HTC on day 1 (Figure 9a), where HTC appears to increase in contrast to the reported studies that show a continuous drop in HTC as the condensation progresses, could be an indication of the unique wettability feature of porous nanochannel LIS. Due to the low thermal conductivity (∼0.6 W/m^–1^ K^–1^) of silicon oil, which acts as a thermal barrier, extra thickness of oil on the surface of freshly prepared LIS is unfavorable for heat transfer. Therefore, HTC is seen to improve because the underlying porous substrate can still retain necessary oil and maintain hydrophobic properties while extra oil on the surface depletes with drop shedding, thus facilitating quicker condensate shedding from the surface. This explains why the average HTC in final 4 h of condensation (2.12 ± 0.02 Wm^–2^ K^–1^) on day 1 was around ∼16% higher than the initial 4 h of HTC and nearly as high as HTC (2.33 ± 0.42 Wm^–2^ K^–1^) of dep-nc-LIS-50 (Figure 7a). The details of temperature acquisition rate and fluctuations in HTC is explained in Supporting Information, note S5. After the first day of the experiment, the LIS sample was kept in an open lab environment for 15 days before condensation was started again on day 17 and continued for 2 additional days. It is important to note that for these experiments, which span over multiple hours, the water level in the vapor generation system was maintained by a temperature-controlled feedback-based pump circuit unlike manual intervention in the case of condensation on depleted LIS. Variation of HTC on nc-LIS-50 for the last 2 days (days 17 and 18) is shown in Figure 9a. Cumulative effect of oil depletion during day 1 experiment, surface contamination, and oil depletion due to gravity over 15 days can be seen in the lower average HTC value of 1.46 ± 0.16 Wm^–2^ K^–1^. Over the total 3 days of condensation on nc-LIS-50, the shedding condensate drop size was observed to increase (Figure 9b), but the nc-LIS surface maintained dropwise condensation, never transitioned to filmwise condensation, and remained hydrophobic (WCA ∼ 104°) at the end of experiments, thus showing the potential applicability of our LIS samples for real-world applications.

## 4 Conclusions

Condensation heat transfer on liquid-infused surfaces (LISs) necessitate seemingly conflicting requirements of high sliding velocity and low lubricant depletion. Although, oil depletion can be mitigated to some extent, it has been established by the reported studies to be omnipresent in lubricant-infused surfaces, eventuating in the gradual decline of heat-transfer performance. In this work, porous nanochannel wick-based lubricant-infused surfaces (nc-LIS) show excellent drop mobility (roll-off angle ∼1°) and demonstrate improved condensation heat-transfer performance in the presence of non-condensable gases. Non-toxic immiscible silicone oil of different viscosities functioned as a lubricating medium in the porous substrate comprising cross-connected buried nanochannels with micropores present at intersections. Based on the comparison of four different silicone oil viscosities, it was observed that higher silicone oil viscosity nc-LIS exhibited prolonged retention on the surface, but the drop sliding velocity was significantly lower compared to nc-LIS with low viscosity silicone oil. nc-LIS using 50 cSt and 500 cSt silicone oil are shown to provide substantial improvement in the condensation heat-transfer coefficient (HTC ∼2.33 kW m^–2^ K^–1^ for 50 cSt and 1.66 kW m^–2^ K^–1^ for 500 cSt) even in depleted conditions, and no significant change (ΔWCA ∼ 3°) in the contact angle was observed for such LIS samples. Improved performance can be attributed to oil being held inside nanochannels and, thus, retained on the surface, due to capillarity and improved adhesion owing to plasma cleaning. The drop size distribution study revealed that more than ∼95% of all drops had a diameter <500 μm as is evident from videos captured at different instances throughout the experiments. Experiments conducted on the flat silicon surface with 50 cSt oil under fresh conditions show a lack of adequate oil on the surface as multiple coalescence of condensate occurs before drop departure which led to only 28% of drops having a diameter <250 μm and 61% of drops with diameter <500 μm. Condensation on fresh nc-LIS with 50 cSt silicone oil for 3 days revealed an improvement in the heat transfer coefficient of 16% from the start of condensation (0–4 h HTC: 1.88 ± 0.11 W m^–2^ K^–1^) toward the end of first 24 h (20–24 h HTC: 2.12 ± 0.02 W m^–2^ K^–1^), as extra oil depletion reduced the thermal barrier for condensation heat transfer while the porous geometry retained the necessary oil to keep the surface hydrophobic. After a 15 day gap during which the sample was kept exposed to ambient conditions, steady-state HTC was attained over the last 2 days of experiments with an average value: 1.46 ± 0.16 Wm^–2^ K^–1^. Over the course of this long experimental duration, the sample maintained dropwise condensation, never transitioned to film-wise condensation, and remained hydrophobic with WCA ∼104° at the end of condensation. We anticipate that the presented LIS preparation approach can be implemented on large-scale porous surfaces for heat transfer-based applications with improved performance even under depleted conditions. Moreover, the design of such system could be tailored to suit the needs of a variety of applications such as drag reduction surfaces or in the medical field to generate inert, non-toxic and non-adhesive surfaces.

## Supporting Information Available

Sample fabrication, characterization, experimental section, sliding velocity plots, contact angle plots, temperature variation during condensation experiments, uncertainty analysis, and material properties (PDF)
nc-LIS kept horizontally under a tap water jet to deplete oil under shear imparted by a running tap water (jet velocity ∼0.4 ms^–1^) for 20 min from a nozzle (MP4)
