# Supplementary material for: Dropwise Condensation in Ambient on a Depleted Lubricant-Infused Surface
Source: ACS Appl Mater Interfaces. 2023 Apr 20;15(17):21679–89. doi: 10.1021/acsami.3c02450 (PMC10165607; doi:10.1021/acsami.3c02450)
Supplement: Supplementary file 1 — am3c02450_si_001.pdf [file am3c02450_si_001.pdf]

Supporting Information: Dropwise Condensation in Ambient on Depleted Lubricant-Infused Surface

Durgesh Ranjan, Maheswar Chaudhary, An Zou, and Shalabh C. Maroo*
Department of Mechanical and Aerospace Engineering, Syracuse University, Syracuse, NY, 13244, United States; *Correspondence: scmaroo@syr.edu

SUPPORTING NOTES

Note S1

Fabrication schematic is shown in Figure S1. Fabrication of porous nanochannel (nc) wicks starts with a 500 μm silicon wafer. Firstly, mark fabrication on the wafer is carried out by photoresist coating, exposing in GCA stepper with suitable reticle followed by baking, developing, and etching. After removing photoresist, a mark on wafer leaves behind which is helpful in alignment during exposure in later stages. Then, Photoresist (thickness ~ 9 μm) was spin coated on the wafer and then exposed using GCA stepper followed by an ammonia diffusion bake for the image reversal. After photoresist development, 15 nm thick chromium (Cr) film obtained deposited by e-beam evaporator (deposition rate 1.1 - 1.3 A/s) was used as adhesion layer on top of which 713 nm thick copper (Cu) film (deposition rate 3.0 - 3.5 A/s) was further deposited (using e-beam evaporator). Then, the photoresist was removed, leaving behind Cr and Cu on silicon wafer then immersed in acetone for 6 hours. This lift-off process created orthogonally connected ridges of sacrificial Cr & Cu. Then, 300 nm thick SiO_2 film was deposited over patterned Cr & Cu layer by plasma enhanced chemical vapor deposition (PECVD). After depositing 300 nm thick SiO_2, SPR 220-3 photo resist was used to obtain a ~ 3 μm coating on the wafer. After baking, GCA stepper was used to expose the sample using custom made reticle (~ 2 μm diameter holes at 10 μm pitch, exposure time 0.5 s) and executing required job file. It is important to note that alignment of reticle is very important while executing exposure such holes are exposed at the intersection of the ridges. After hard bake and development wafer with exposed SiO_2 (at ~ 2 μm diameter holes) undergoes dry etching followed by photoresist removal in hot bath and plasma strip resist. The wafer was immersed in Cr & Cu etchant to remove sacrificial Cr & Cu to form the nanochannels which are interconnected orthogonally and open to environment via pores (~ 2 μm holes). Wafer is cut in to required dimension of sample using dicing saw.

Note S2

In the FT-IR spectrum of nc-dep LIS (Figure S2), the absorption peaks located at 2962 cm^{-1} and 2904 cm^{-1} are due to the asymmetric and symmetric stretching vibration of C-H in methyl and methylene groups present in silicone oil, respectively [1] and the peaks appeared at 1413 cm^{-1} and 666 cm^{-1} can be attributed to the bending vibration of C-H bonds [2] due to the remaining non-reacted precursors or the presence of adsorbed fats or contaminants on the surface of the material. The peaks centered at 1259 cm^{-1}, 1085 cm^{-1}, and 1016 cm^{-1} can be related to the stretching vibration of Si-OH [3] and asymmetric and symmetric stretching vibration of Si-O-Si, respectively [4,5]. The absorption peak centered at 794 cm^{-1} is due to the bending vibration of the Si-O-Si bonds [6].

Note S3

The velocity measurement was performed by analyzing the drop sliding frames recorded by the high-speed camera. To visualize the drop, a solution of sodium fluorescein was prepared (0.1 gm in 1 liter of deionized water)[7] and a custom written MATLAB algorithm was used to track the motion of water drop as shown in Figure S3. The images obtained from the high-speed camera were counter rotated by same angle as that of inclination of sample to make the drop motion appear horizontal during image processing. Then, after cropping out the required area in the image, it is converted into binary i.e., black & white image. This enabled easy tracking of the contour front of the drop as the white pixels on the drop would always have value 1. In Figure S3, P1 (point on drop front at the beginning of drop sliding) and P2 (point on drop front after time "t" of drop sliding) shows the location of front being tracked. The difference between pixel location along with calibrated pixel length (i.e., number of pixels in 1 mm denoted as "p") would give the distance travelled by the drop. Elapsed time (t) between frames was obtained from number of frames (nf) and captured frame rate (fps) of video. Velocity was obtained as,

$$\text{Velocity} = \frac{(P_1 - P_2) * fps}{p * nf}$$

Equation S1

Note S4

To capture the temperature variation at the outlet we conducted an experiment with 4 thermocouples instead of 1 thermocouple. Figure S6a shows the 4 thermocouples (T1-T4) inserted (1.5 cm from the cold plate) at different depths, and two more thermocouples (T5-T6, Figure S6b) inserted at 5 cm from the previous 4 thermocouples. The distance of four thermocouple tip from the inside surface of the tube are: 0.49 mm, 1.33 mm, 1.55 mm, 2.89 mm. This distance has been calculated using MATLAB based on the RBG values of pixels at the tip and on the inside of the tube. The accuracy corresponding to 1 pixel was 15 μm. The attachment of the thermocouples arrangement to the cold plate is shown in Figure S5. The tube inside cold plate is shown as dashed line in Figure S5. Condensation experiment was carried out with primary interest being the temperature readings at the outlet. Figure S6d-e show the variation of temperature recorded by all six thermocouples. The maximum difference between any two thermocouples among T1-T4 is < 2ºC. The temperature readings of T5 and T6 which would see some mixing due to sharp contraction show maximum difference < 2ºC. This maximum temperature difference of less than 2ºC is within the maximum error listed in the experiments (Table S2). Moreover, for all our experimental data and analysis (as in the manuscript), we used data from one thermocouple which was placed near the center of the outlet tube, which would underestimate (that too only by < 2ºC) the outlet temperature rather than overestimating it; thus, the results in our work are conservatively presented. Hence, we can conclude that temperature variation within the thermal boundary layer is insignificant for our experiments and analysis. In fact, similar arrangements of thermocouples has been reported in literature to measure the temperature[8–10].

Note S5

Uncertainty related with temperature and humidity was taken as the standard deviation in data recorded during the condensation experiment. Condensation heat transfer coefficient (HTC) was calculated at each data point in temporal domain and related standard deviation in HTC is due to fluctuations in temperatures during experiments. The uncertainty calculation for condensation heat flux (q_c'') is obtained using following equations:

$$q_c'' = h_c \, \Delta T_{sub}$$

Equation 1

$$\Delta q_c'' = h_{c,avg}\,(\Delta T_{sub,avg})\sqrt{\left(\frac{\Delta h_c}{h_{c,avg}}\right)^2 + \left(\frac{\Delta(\Delta T_{sub})}{\Delta T_{sub,avg}}\right)^2}$$

Equation 2

Where, q_c'' is condensation heat flux (Wm^{-2}), h_c is condensation heat transfer coefficient ($Wm^{-2}K^{-1}$), ΔT_{sub} is the subcooling (temperature difference between nc-dep surface temperature and condensation chamber temperature, ºC), $\Delta q_c''$ is uncertainty associated with condensation heat flux (Wm^{-2}), Δh_c is uncertainty associated with condensation heat transfer coefficient ($Wm^{-2}K^{-1}$), $\Delta(\Delta T_{sub})$ is uncertainty associated with subcooling, ºC). Mean values and uncertainty associated with various parameters are given in Table S2.

Temperature collection rate during condensation on fresh porous nanochannel sample is given below:

Day 1-2: (a) First 2 hours: 30 data per hour, remaining 22 hours: 10 data per hour, (b) day 17-18: 30 data per hour for 48 hours.

The acquisition rate captures the fluctuations in the temperature and hence the deviation of heat transfer coefficient (HTC) from mean value; the higher the rate of acquisition, higher the sudden fluctuation in HTC and vice-versa. However, trend of mean HTC would not be affected as it is governed by surface wettability characteristics rather than sudden fluctuations in ambient and cold thermal fluid temperature. Moreover, automatic control of water level in vapor generation system will result in less variation in ambient temperature (Figure S6) as in case of condensation on fresh LIS on porous nanochannel sample than a manually maintained water level (Figure S8) as in case of condensation on depleted sample.

Note S6

Drop size distribution for flat silicon surface is shown in Figure S7. It was found that percentage of drops having diameter < 250 µm at the start and towards the end of condensation experiment was ~ 41% and ~ 28% respectively. For drops having diameter < 500 µm, it was ~ 80% and ~ 60% respectively.

SUPPORTING FIGURES

Supporting Figure S1

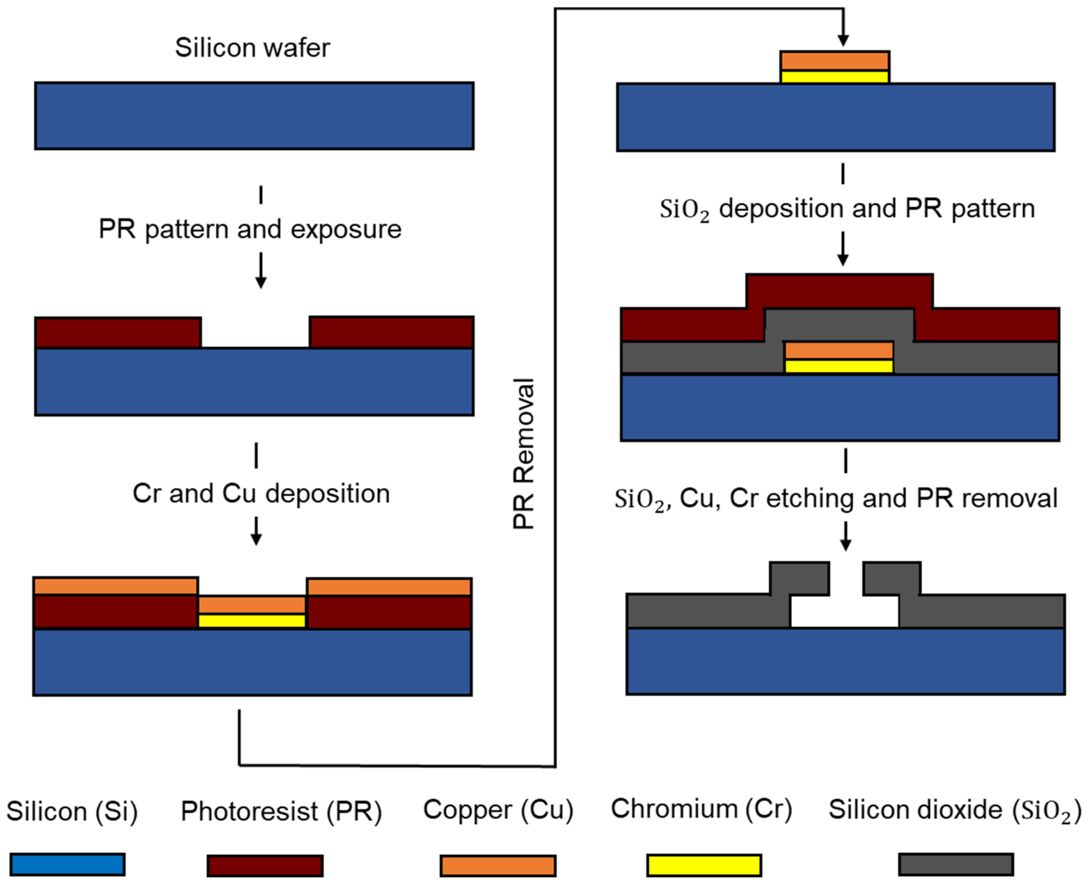

Figure S1. Porous nanochannel wick fabrication procedure.

Supporting Figure S2

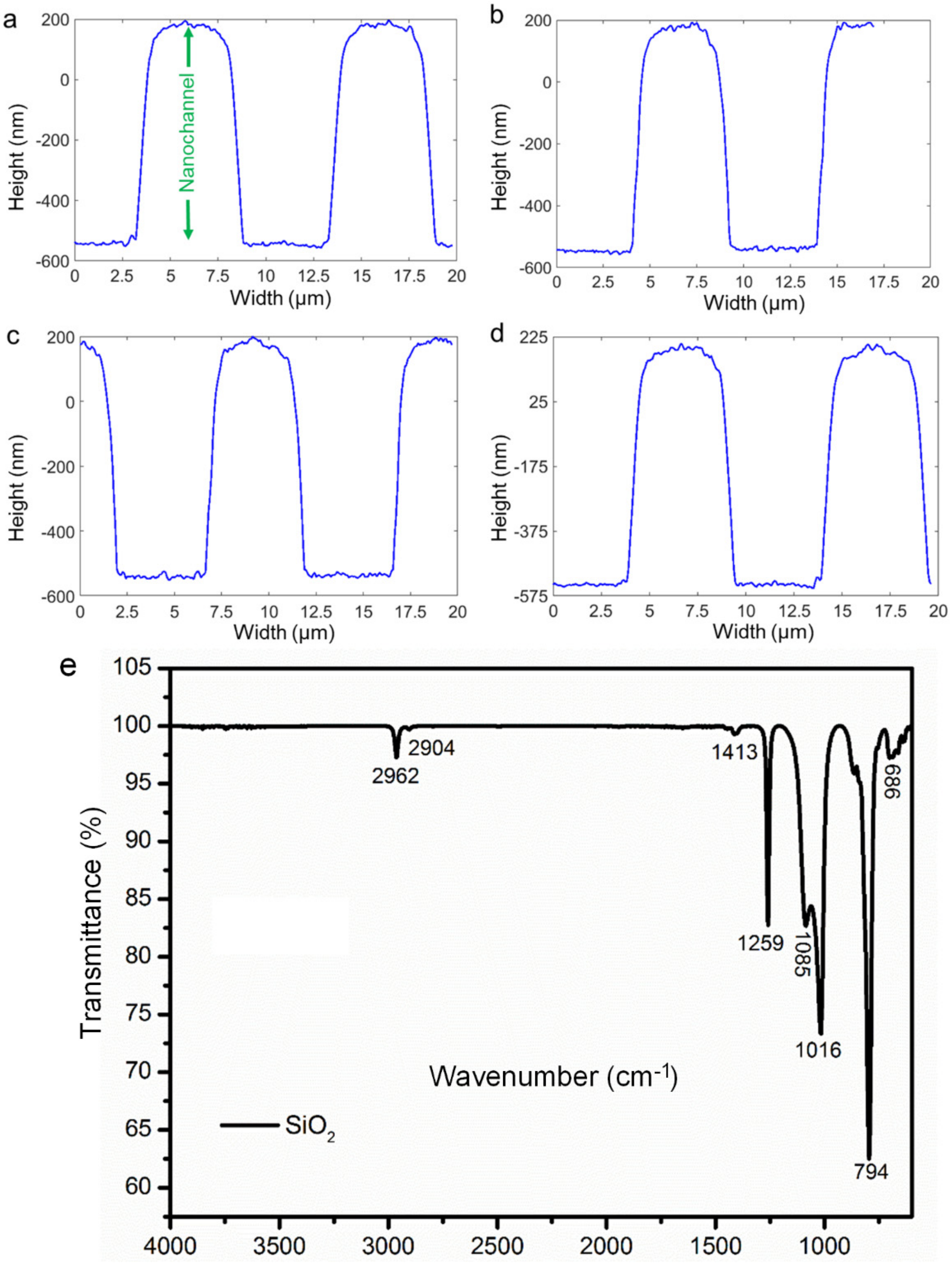

Figure S2. (a-d) Variation of the nanochannel height across width for multiple locations. The height and width were found to be 729 ± 8 nm and 5.68 ±0.87 µm, respectively, (e) Fourie- transform infrared spectroscopy (FTIR) of water jet shear induced depleted nc-LIS.

Supporting Figure S3

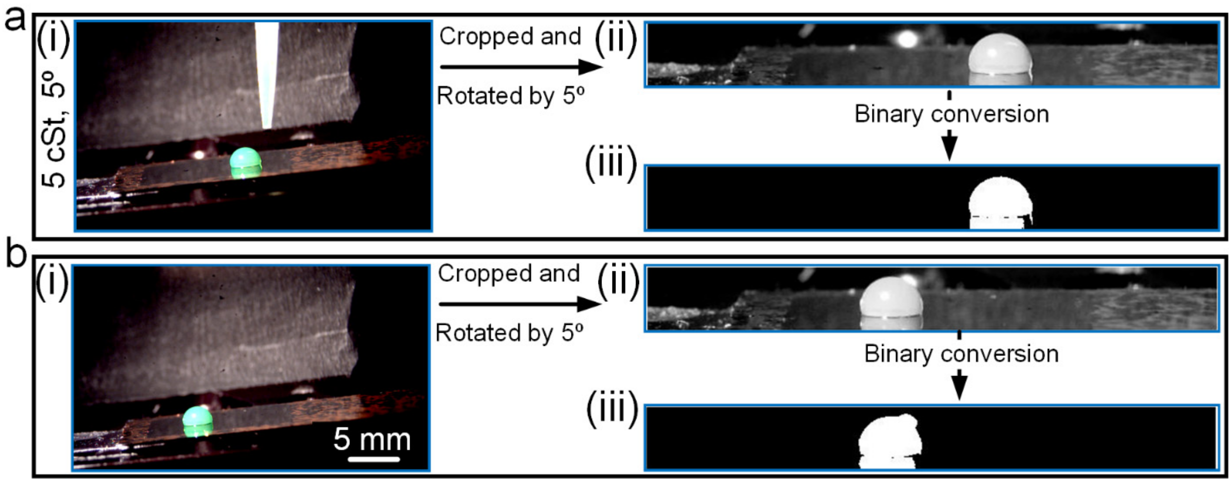

Figure S3. Drop sliding velocity measurement procedure. (a) (i)Initial drop position at the start of sliding, (ii) rotation of image by an angle same as that of sample inclination angle followed by cropping and grey scale conversion, (iii) binary (black and white) conversion (b) (i) final drop position after sliding for some time "t" (ii) rotation of image by an angle same as that of sample inclination angle followed by cropping and grey scale conversion, (iii) binary (black and white) conversion

Supporting Figure S4

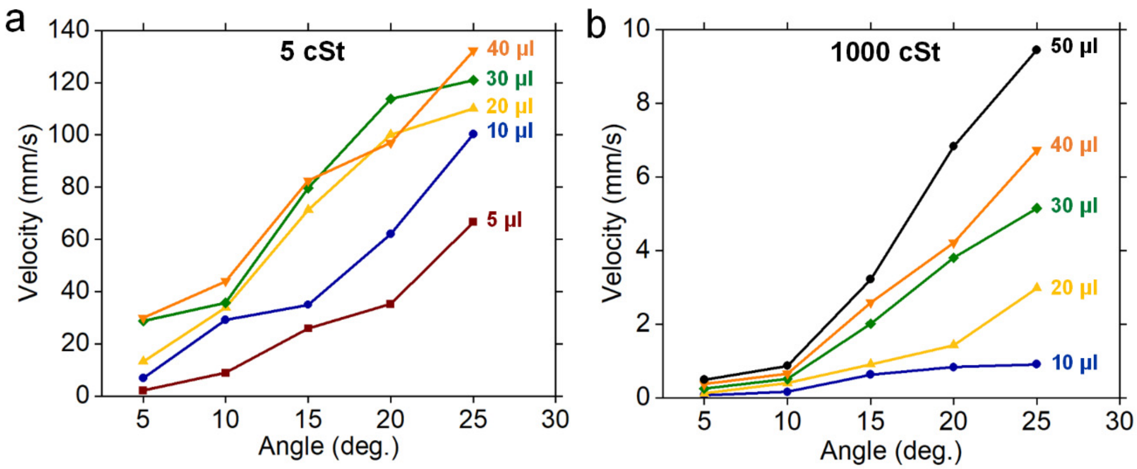

Figure S4. Sliding velocity (V_s) for water drop of various volume on freshly-prepared nanochannel lubricant infused surface (nc-LIS): (a-b) variation of water (volume: 5 µl – 50 µl) droplet velocity for different angle of inclination and all four prepared nc-LIS with different silicone oil viscosity (5 cSt, 1000 cSt).

Supporting Figure S5

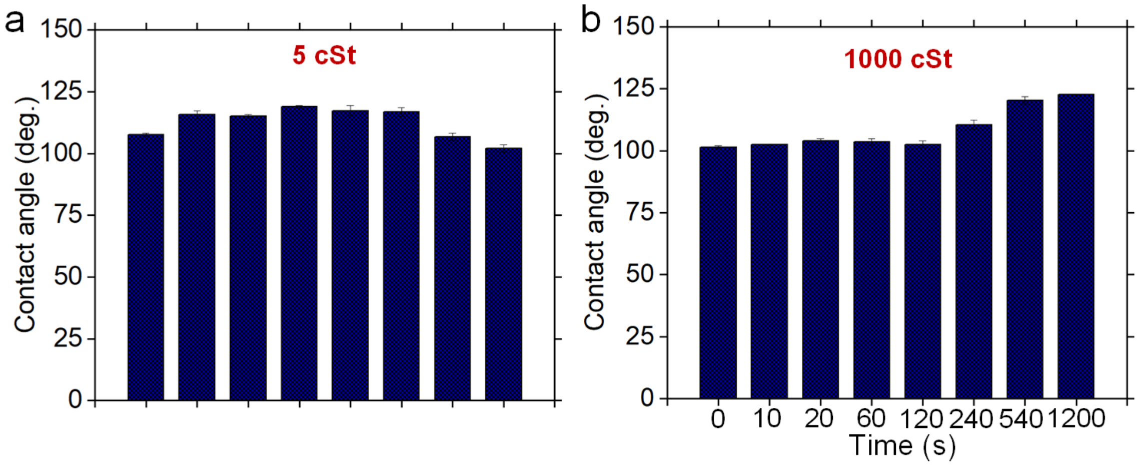

Figure S5. Variation in water contact angle observed during tap water shear depletion of fresh nc-LIS for prepared samples having different silicone oil viscosity (5 cSt, 1000 cSt).

Supporting Figure S6

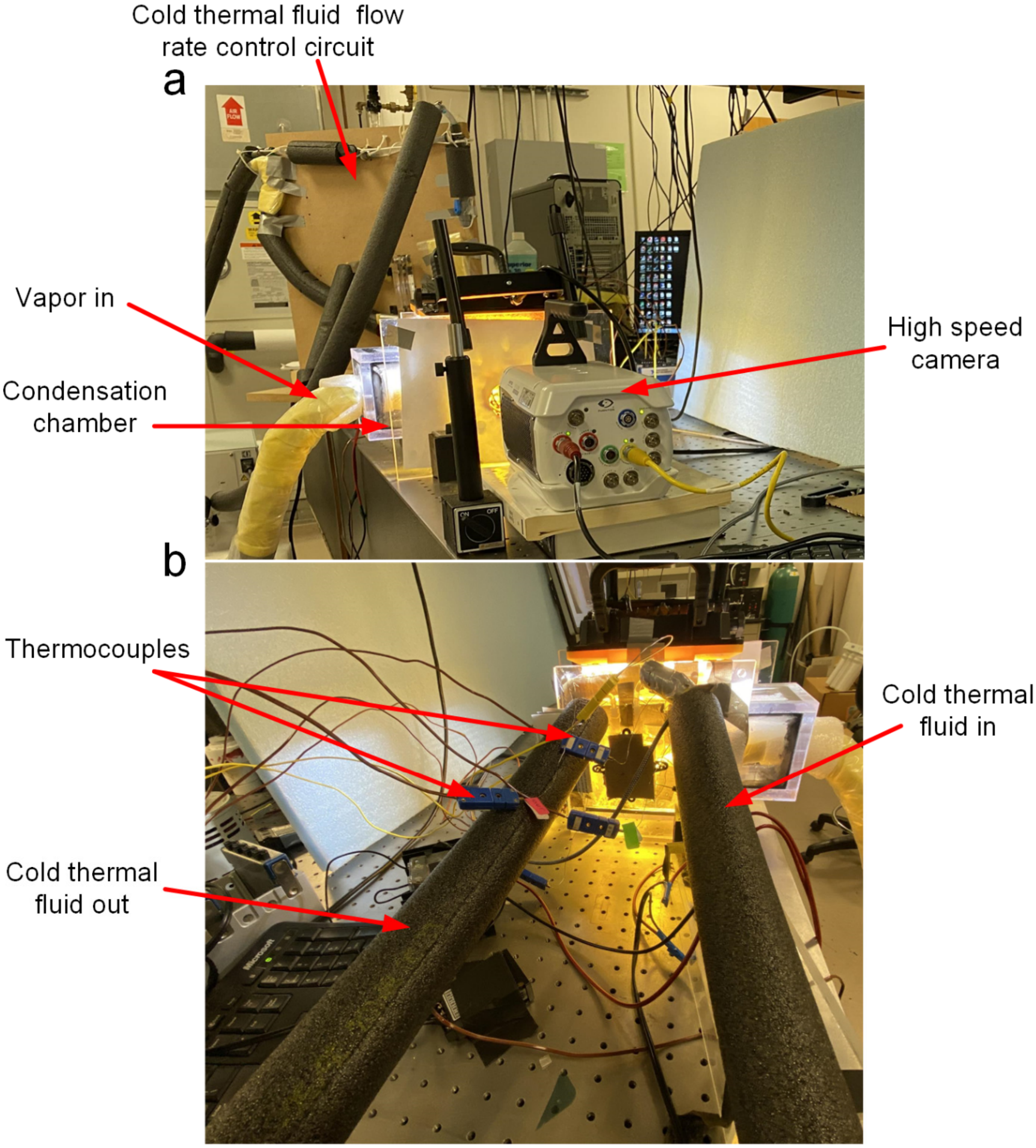

Figure S6. Condensation experimental setup (a) components of the setup, with cold thermal fluid circuit board having height of 2 ft, and (b) view from the fluid flow rate control circuit board towards condensation chamber.

Supporting Figure S7

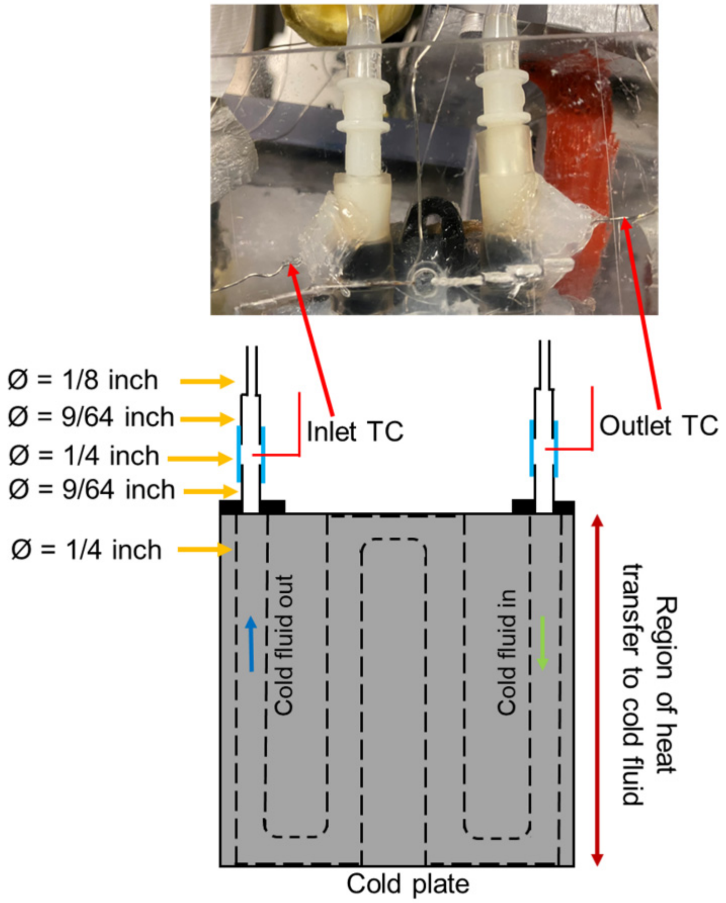

Figure S7. Thermocouple placement at the thermal fluid inlet and outlet of cold plate and the geometry of the pipes at inlet and outlet.

Supporting Figure S8

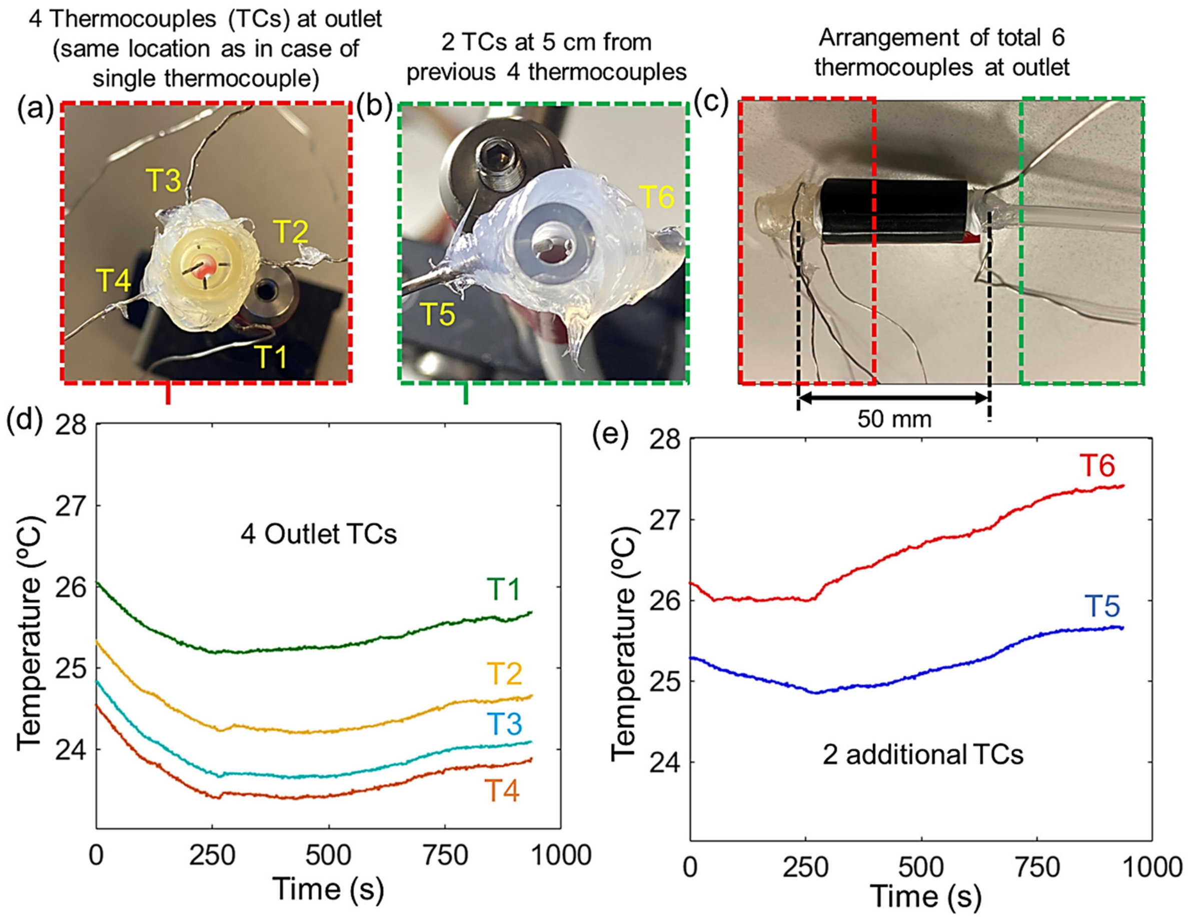

Figure S8. Experimental arrangements for observing the effect of thermal boundary layer at the outlet of the cold plate (a) four thermocouples (T1-T4) located at the 1.5 cm from the cold plate (b) two additional thermocouples (T5-T6) located at the 5 cm from the previous four thermocouples (c) arrangement of all six thermocouples in the outlet pipe. (d-e) variation of temperature recorded by the six thermocouples (T1-T6).

Supporting Figure S9

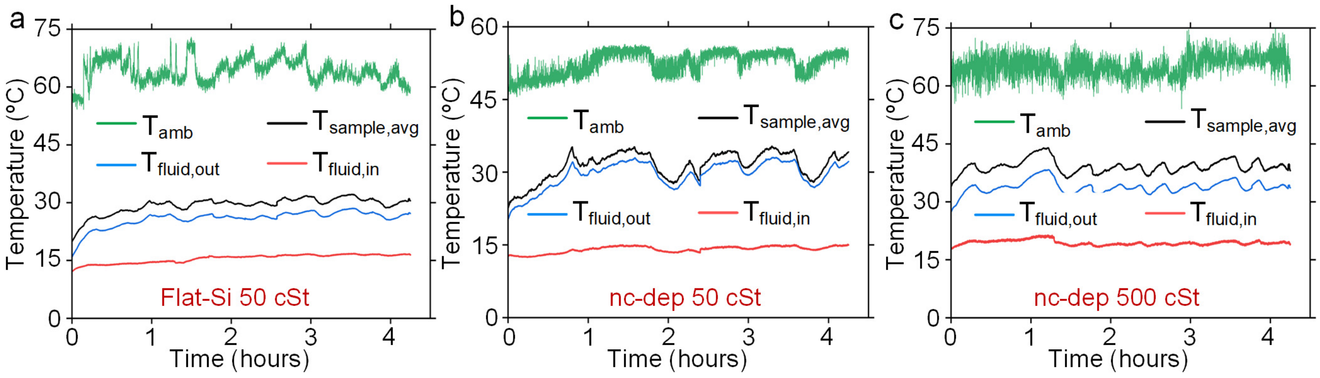

Figure S9. Variation of ambient chamber temperature (T_{amb}), average sample temperature ($T_{sample,avg}$), thermal fluid at temperature cold plate outlet ($T_{fluid,out}$) and thermal fluid at cold plate temperature inlet ($T_{fluid,in}$) during condensation for: (a) LIS on flat silicon surface with 50 cSt oil, (b) depleted LIS on porous nanochannel with 50 cSt oil, and (c) depleted LIS on porous nanochannel with 500 cSt oil.

Supporting Figure S10

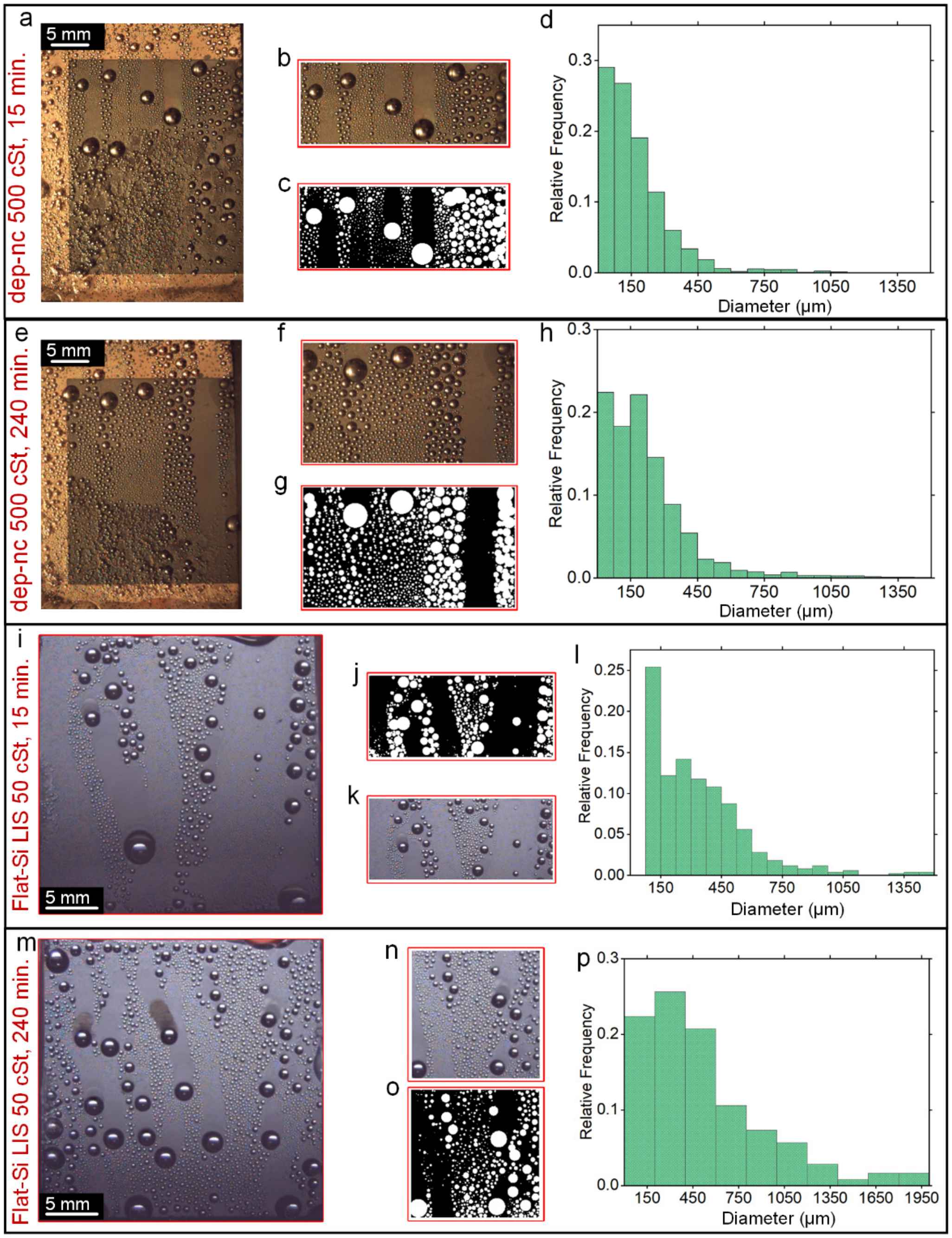

Figure S10. Drop size distribution for a selected region on depleted liquid infused surface (dep-nc-LIS) and flat silicon (flat Si) liquid infused surface (LIS) sample during experiment for (a-d) dep-nc-LIS 500 cSt after 15 minutes of condensation, (e-h) dep-nc-LIS 500 cSt after 240 minutes of condensation, (i-l) flat-Si 50 cSt after 15 minutes of condensation, (m-p) flat-Si 50 cSt after 240 minutes of condensation.

Supporting Figure S11

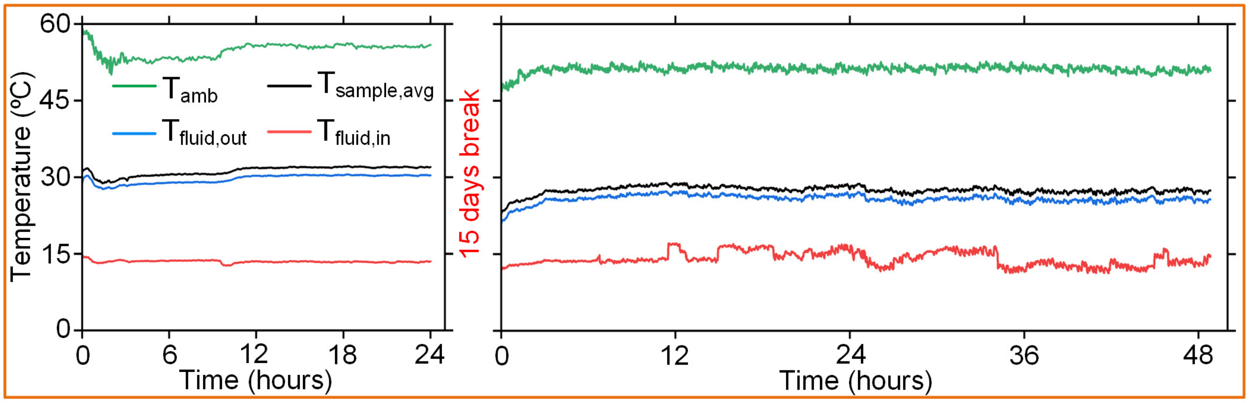

Figure S11. Variation of ambient chamber temperature (T_{amb}), average sample temperature ($T_{sample,avg}$), thermal fluid temperature at cold plate outlet ($T_{fluid,out}$) and thermal fluid temperature at cold plate inlet ($T_{fluid,in}$) during condensation for freshly prepared LIS on porous nanochannel with 50 cSt oil

Table S1. Physical properties of materials used in current study.

Material	Density (kgm^{-3})	Viscosity (centistokes) (cSt)	Surface Tension (mNm^{-1})	Specific Heat Capacity ($kJ\ kg^{-1}K^{-1}$)
Silicone oil	918	5	19	N/A
	950	50	21	N/A
	971	500	21	N/A
	971	1000	21	N/A
Water-glycol (50:50)	1085	N/A	N/A	3.44

Table S2. Errors and uncertainties related to various parameters.

Parameter (unit)	Mean Value	Standard Deviation/ Uncertainty
ΔT_w (ºC): flat Si-LIS 50 cSt	10.3	1.2
ΔT_w (ºC): nc-dep-LIS 50 cSt	16.5	2.1
ΔT_w (ºC): nc-dep-LIS 500 cSt	14.4	1.1
ΔT_w (ºC): nc-LIS 50 cSt (day 1-2) : 24 hrs	16.2	1.3
ΔT_w (ºC): nc-LIS 50 cSt (day 17-18): 48 hrs	11.7	1.2
ΔT_{sub} (ºC): flat Si-LIS 50 cSt	34.74	3.47
ΔT_{sub} (ºC): nc-dep-LIS 50 cSt	21.1	1.8
ΔT_{sub} (ºC): nc-dep-LIS 500 cSt	25.9	2.6
ΔT_{sub} (ºC): nc-LIS 50 cSt (day 1-2): 24 hrs	23.5	2.3
ΔT_{sub} (ºC): nc-LIS 50 cSt (day 17-18): 48 hrs	23.6	0.7
h_c ($Wm^{-2}K^{-1}$): flat Si-LIS 50 cSt	0.89	0.16
h_c ($Wm^{-2}K^{-1}$): nc-dep-LIS 50 cSt	2.33	0.42
h_c ($Wm^{-2}K^{-1}$): nc-dep-LIS 500 cSt	1.66	0.25
h_c ($Wm^{-2}K^{-1}$): nc-LIS 50 cSt (day 1-2): 24 hrs	2.07	0.14
h_c ($Wm^{-2}K^{-1}$): nc-LIS 50 cSt (day 17-18): 48 hrs	1.46	0.16
q_c'' (kWm^{-2}): flat Si-LIS 50 cSt	30.88	6.29
q_c'' (kWm^{-2}): nc-dep-LIS 50 cSt	49.18	9.76
q_c'' (kWm^{-2}): nc-dep-LIS 500 cSt	42.95	7.70
q_c'' (kWm^{-2}): nc-LIS 50 cSt (day 1-2): 24 hrs	47.81	5.64
q_c'' (kWm^{-2}): nc-LIS 50 cSt (day 17-18): 48 hrs	34.67	3.89

Supporting Movie Captions

Movie S1 Description: Water jet shear depletion of nanochannels LIS.

REFERENCES:

(1) Vanitha, V.; Hemalatha, S.; Pushpabharathi, N.; Amudha, P.; Jayalakshmi, M. Fabrication of Nanoparticles Using Annona Squamosa Leaf and Assessment of Its Effect on Liver (Hep G2) Cancer Cell Line. *IOP Conf. Ser. Mater. Sci. Eng.* **2017**, *191* (1). https://doi.org/10.1088/1757-899X/191/1/012010.

(2) Kunjiappan, S.; Chowdhury, R.; Bhattacharjee, C. Isolation and Structural Elucidation of Flavonoids from Aquatic Fern Azolla Microphylla and Evaluation of Free Radical Scavenging Activity ISOLATION AND STRUCTURAL ELUCIDATION OF FLAVONOIDS FROM AQUATIC FERN AZOLLA. *Int. J. Pharm. Pharm. Sci.* **2013**, *5* (3), 743–749.

(3) Ahmadizadeh Shendy, S.; Babazadeh, M.; Shahverdizadeh, G. H.; Hosseinzadeh-Khanmiri, R.; Es'haghi, M. Synthesis of the Quinazolinone Derivatives Using an Acid-Functionalized Magnetic Silica Heterogeneous Catalyst in Terms of Green Chemistry. *Mol. Divers.* **2021**, *25* (2), 889–897. https://doi.org/10.1007/s11030-020-10033-1.

(4) Saravanan, S.; Dubey, R. S. Synthesis of SiO2 Nanoparticles by Sol-Gel Method and Their Optical and Structural Properties. *Rom. J. Inf. Sci. Technol.* **2020**, *23* (1), 105–112.

(5) Javidparvar, A. A.; Ramezanzadeh, B.; Ghasemi, E. Effect of Various Spinel Ferrite Nanopigments Modified by Amino Propyl Trimethoxy Silane on the Corrosion Inhibition Properties of the Epoxy Nanocomposites. *Corrosion* **2016**, *72* (6), 761–774. https://doi.org/10.5006/2021.

(6) Tsamesidis, I.; Lymperaki, E.; Egwu, C. O.; Pouroutzidou, G. K.; Kazeli, K.; Reybier, K.; Bourgeade-Delmas, S.; Valentin, A.; Kontonasaki, E. Effect of Silica Based Nanoparticles against Plasmodium Falciparum and Leishmania Infantum Parasites. *J. Xenobiotics* **2021**, *11* (4), 155–162. https://doi.org/10.3390/jox11040011.

(7) Sherstad, E. M. Use of Fluorescein To Measure the Composition of Waterdrop Splash. **1971**, No. 4, 1020–1023.

(8) Zhang, T. Y.; Mou, L. W.; Zhang, J. Y.; Fan, L. W.; Li, J. Q. A Visualized Study of Enhanced Steam Condensation Heat Transfer on a Honeycomb-like Microporous

Superhydrophobic Surface in the Presence of a Non-Condensable Gas. *Int. J. Heat Mass Transf.* **2020**, *150*, 119352. https://doi.org/10.1016/j.ijheatmasstransfer.2020.119352.

(9) Tsuchiya, H.; Tenjimbayashi, M.; Moriya, T.; Yoshikawa, R.; Sasaki, K.; Togasawa, R.; Yamazaki, T.; Manabe, K.; Shiratori, S. Liquid-Infused Smooth Surface for Improved Condensation Heat Transfer. *Langmuir* **2017**, *33* (36), 8950–8960. https://doi.org/10.1021/acs.langmuir.7b01991.

(10) Hu, H. W.; Tang, G. H.; Niu, D. Experimental Investigation of Condensation Heat Transfer on Hybrid Wettability Finned Tube with Large Amount of Noncondensable Gas. *International Journal of Heat and Mass Transfer*. 2015, pp 513–523. https://doi.org/10.1016/j.ijheatmasstransfer.2015.02.006.
